# Behaviour change techniques in eHealth interventions for older, frail, or sarcopenic adults: A systematic review and meta-analysis

**DOI:** 10.1177/20552076261473804

**Published:** 2026-07-28

**Authors:** Ayushi Omkar, Jennifer K. Bertrand, Chikku Sadasivan, Emma Osness, Ben Vandermeer, Xiaoxu Ding, Liz Dennett, Julian Mansour, Victor E. Ezeugwu, Ashley Hyde, Puneeta Tandon

**Affiliations:** 1 Department of Medicine, Division of Gastroenterology (Liver Unit), 12357 University of Alberta, Edmonton, AB, Canada; 2 Department of Medicine, 12357 University of Alberta, Edmonton, AB, Canada; 3 Geoffrey and Robyn Sperber Health Sciences Library, 3158 University of Alberta, Edmonton, AB, Canada; 4 Faculty of Rehabilitation Medicine, 70412 University of Alberta, Edmonton, AB, Canada; 5 Faculty of Nursing, 3158 University of Alberta, Edmonton, AB, Canada

**Keywords:** eHealth, digital health, digital intervention, physical activity, behaviour change, behaviour change techniques, frailty, older adults, systematic review, meta-analysis

## Abstract

**Background:**

Behaviour change techniques (BCTs) are key components of eHealth physical activity interventions. They may be especially important for older adults and individuals with frailty or sarcopenia, who often face additional barriers to sustaining physical activity. This review aimed to identify BCTs used in eHealth physical activity trials and examine which techniques are associated with greater effectiveness.

**Methods:**

Six databases (MEDLINE, Embase, CINAHL, CENTRAL, PsycINFO, and Scopus) were searched for randomized controlled trials (RCTs) published up to 2024. Eligible studies evaluated eHealth physical activity interventions delivered to older adults (mean age ≥60 years) or adults with frailty or sarcopenia. Trials delivered exclusively through virtual reality, wearable devices, or telephone-based modalities were excluded. Random-effects meta-analysis estimated pooled effects on physical activity, with subgroup analyses examining differences according to BCTs reported and behavioural theory. Completion, adherence, and adverse events were summarized descriptively.

**Results:**

Eighty-seven studies met inclusion criteria, and 53 contributed to the meta-analysis. eHealth interventions produced a small positive effect on physical activity (standardized mean difference = 0.22, 95% confidence interval: 0.13 to 0.31). Completion was generally high and adherence moderate, with few reported adverse events. The most frequently used BCTs were feedback on behaviour, goal setting, social support, self-monitoring, and prompts/cues. Among these BCTs, all were non-significant, with social support (unspecified) having a p=0.05.

**Conclusions:**

eHealth interventions yield modest improvements in physical activity among older adults, although evidence specific to frailty and sarcopenia remains limited. Further research is needed to better understand the role of social support and other BCTs in eHealth physical activity interventions among older adults.

**Systematic review registration:**

PROSPERO CRD42025641608.

## Introduction

Physical activity is any movement generated by skeletal muscles that requires energy; it includes a range of activities, including exercise,^1^ and is important for preventing and managing various noncommunicable diseases.^2^ Regular physical activity is linked to improved cardiovascular health, muscular strength, cognitive function, and overall quality of life.^3–6^ Despite these benefits, activity levels remain low, particularly among high-risk populations. An estimated 31% of individuals over age 60 do not meet recommended physical activity guidelines. This number increases to 60-75% in those with sarcopenia or frailty.^7–10^ While these statistics highlight the need for targeted interventions to increase physical activity in these populations, several barriers exist, including low motivation, difficulty accessing in-person programs, and limited social support.^11–13^ The remote support, tailored feedback, and flexible participation of an eHealth platform may offer a scalable solution to address these challenges and increase engagement with physical activity.

eHealth is a broad term for the use of digital technologies to deliver healthcare services, including mobile apps, websites, wearable devices, and telemedicine, enabling remote healthcare delivery.^14^ In the context of physical health interventions, eHealth offers several benefits, such as personalization, cost-effectiveness, and improved accessibility.^15–17^ This approach can help expand access to physical activity programs while maintaining or enhancing their effectiveness.^18^

While eHealth interventions for physical activity show potential benefits, they also pose challenges for older adults and those with frailty or sarcopenia. These challenges include limited technological skills, physical or cognitive barriers to device use, reluctance to learn new technologies, and a lack of user-friendly interfaces.^19–21^ To better understand how to support behaviour change in these increased risk populations, it is useful to explore the behaviour change techniques (BCTs) and theories that enhance adherence and acceptance.^22^ BCTs, as defined by Michie’s Behaviour Change Technique Taxonomy (BCTTv1), are the active ingredients of interventions and play a key role in initiating and sustaining behaviour change.^23^ They are essential for promoting sustainable, long-term changes, such as increased physical activity and improved dietary habits.^24,25^ They can also increase the understanding of the mechanisms driving behaviour change, leading to more effective interventions.^26^ Although several systematic reviews have provided detailed assessments of BCTs in promoting non-eHealth physical activity,^25,27,28^ existing reviews of eHealth interventions in older, frail, or sarcopenic adult populations have offered limited BCT assessment.^18,29,30^

## Methods

### Study design

A systematic review was conducted to identify the number, type, and category of BCTs used in randomized controlled trials of eHealth physical activity interventions in older adults (mean age ≥ 60 years), or adults with frailty or sarcopenia. The format of this review follows the Preferred Reporting Items for Systematic Reviews and Meta-Analysis (PRISMA) guidelines.^31^ The PRISMA 2020 checklist is provided in Supplemental File 1. This review was registered in PROSPERO (CRD42025641608).

### Search strategy

A comprehensive search for RCTs was performed across six databases (MEDLINE (Ovid), Embase (Ovid), CINAHL (EBSCOhost), CENTRAL (Wiley), PsycINFO (Ovid), and Scopus (Elsevier)) from inception to October 8, 2024. No date or other limits were applied to ensure thorough coverage. A health sciences librarian (LD) assisted with the search. The search terms for each database are listed in Supplemental File 2. We also conducted a hand search of reference lists in relevant review articles to identify potentially eligible studies.

### Eligibility criteria

Identified articles were evaluated based on the established inclusion and exclusion criteria using the PICO framework, with study design added as recommended by the Cochrane Handbook.^32^

*Population-* Inclusion required study participants to be one of: (i) older adults (study mean or median age ≥ 60 years), or (ii) adults (≥18 years of age) with frailty or sarcopenia.

*Intervention-* Eligible eHealth interventions focused on behaviour change and included a physical activity component. Interventions delivered through text messages or video calls were included, as these channels provide structured digital communication and may incorporate interactive or tailored elements. Interventions delivered exclusively through wearable devices or telephone delivery were excluded, as these approaches typically involve passive monitoring or episodic verbal contact rather than sustained engagement with a digital platform. However, studies that incorporated wearable devices or telephone support (e.g., telephone reminders, guided activities, or coaching) as a supplementary component of a broader eHealth intervention were eligible for inclusion, provided that a digital platform served as the primary mode of intervention delivery. Virtual reality–based (VR) interventions were also excluded, as VR represents a distinct and emerging modality that does not align with the app and web-based eHealth platforms evaluated in this review.

*Comparator-* Eligible comparators include no eHealth intervention, usual care, or a less advanced eHealth intervention.

*Outcomes-* (i) the number and type of behaviour change categories and techniques used, as seen in the BCT Taxonomy (v1); (ii) whether studies reported using a behaviour change theory as part of their intervention development; (iii) the reported study adherence and completion rates, and clinical effectiveness for each study’s primary outcome measures; (iv) the safety measures and the type/frequency of adverse events in each study.

*Study Design-* RCTs in all languages were eligible for inclusion.

### Study selection

All identified articles were uploaded to Covidence for screening and management.^33^ After removing duplicates, titles and abstracts were independently screened by two reviewers. Full texts were then independently screened by two reviewers. Any discrepancies were resolved through discussion or with a third reviewer.

### Data extraction

Consistent with the Cochrane Handbook of Systematic Reviews of Interventions,^34^ two trained reviewers (AO, EO) extracted data into a predefined template on Covidence. Each reviewer completed five initial extractions to ensure all relevant results were collected. BCTs were coded for both intervention and control groups using Michie’s Behaviour Change Technique Taxonomy (BCTTv1).^23^ After finishing the online BCT Taxonomy (v1) training,^35^ a single reviewer (AO) coded all BCTs for each study to ensure consistency. A second independent reviewer (XD, VE, AH, PT) performed complete duplicate BCT coding of all studies, and discrepancies were resolved through discussion until consensus was reached. Techniques were coded for both control and intervention groups in each RCT to identify techniques shared across study arms. Once all study arms were coded, any BCTs present in both control and intervention were removed. The number and types of BCT categories used in each study were also recorded.^23^ All collected BCT data was reviewed by a second reviewer (VE, PT, AH, XD), along with outcome data (CS, EO).

Demographic characteristics, such as sex, age, health status, race, education level, body mass index (BMI), comorbidities, and technology proficiency, were collected. Intervention characteristics, including eHealth delivery modes, intervention duration, and targeted behavioural domains, were also extracted. If reported, intention-to-treat data were used.

Clinical effectiveness was evaluated based on the primary outcome(s) of each study. If a clear primary outcome was not reported, the study was excluded from analysis. We recorded the mean and standard deviation (SD) at baseline and endpoint for each available outcome and grouped all data into continuous, ordinal, and binary categories. Ordinal outcomes were treated as continuous variables. Studies without control data were excluded. We also extracted data on study completion, adherence, and the frequency and severity of adverse events, as described in the data synthesis.

### Data synthesis

#### Behaviour change techniques

BCTs and BCT categories used in the intervention arm of each study were described by identifying the most commonly used techniques and categories across interventions. BCTs were coded for both intervention and control groups using Michie’s Behaviour Change Technique Taxonomy (BCTTv1).^23^ To accurately assess intervention effectiveness, only BCTs unique to the intervention arm were included, as techniques also delivered to control participants could not be attributed specifically to the intervention. This approach informed subsequent subgroup analyses examining the relationship between BCT use and clinical effectiveness.

#### Clinical effectiveness

For the purposes of this review, clinical effectiveness refers to the effects of interventions across the range of outcomes reported in the included studies. Given the heterogeneity of outcome measures, findings were summarized descriptively and grouped by a single independent reviewer using the COMET outcome taxonomy to provide a consistent structure for organizing the different measures reported across studies.^36^ Outcomes were first classified according to the COMET core areas: physiological and clinical outcomes, life impact, resource use, and adverse events. They were then assigned to one of six domains for this review: physiological and clinical outcomes, behavioural outcomes, physical functioning and fitness, psychosocial and quality-of-life outcomes, feasibility and resource-use outcomes, and safety outcomes. This approach ensured consistent outcome classification across studies. All allocations were verified by a second reviewer, with any disagreements resolved through discussion.

For studies that could not be pooled due to heterogeneity in interventions, populations, or outcomes, a narrative synthesis was conducted. This synthesis summarized the findings by type of BCT used and the outcomes measured, including study adherence, study completion, and the clinical effectiveness of the primary outcomes. BCTs were categorized according to the BCT Taxonomy (v1),^23^ and patterns or associations were presented in both tables and written descriptions.

Due to the substantial clinical heterogeneity among primary outcomes, we standardized the data by converting all outcomes (e.g., means, odds-ratios) into effect sizes to conduct a meta-analysis. For continuous outcomes, baseline and endpoint means and standard deviations (SDs) were extracted for each study. When SDs were not reported, they were calculated using available data such as p-values, 95% confidence intervals (CI), or standard errors. If none of these were provided, the SD was estimated from the interquartile range. A within-study correlation of 0.5 was assumed for SD estimation. Mean differences were then used to compute Hedges’ g standardized mean differences (SMDs) for each outcome.^37^ To maintain consistency and ease of interpretation of effect sizes, a positive sign (+) was assigned when the tailored condition outperformed the comparison condition, and a negative sign (–) indicated the opposite.

For RCTs that included multiple eligible intervention groups, we combined the effect sizes across the groups to represent a single pooled intervention group in the meta-analysis. This ensured that each study contributed only one comparison to the analysis, avoiding duplication of participant data. Similarly, for studies reporting multiple primary outcomes, we pooled the SMDs to generate one composite estimate so that each study contributed only one effect size to the meta-analysis. The pooled effect sizes were calculated using a validated calculator based on the Cochrane Handbook formulas for combining groups with continuous outcomes.^38^ For binary outcomes (e.g., adherence, study completion rates), odds ratios (ORs) with 95% CIs were calculated. When necessary (i.e., when there were 0 events for one of the comparisons), a continuity correction of 0.5 was added to both the number of events (n) and sample size (N) for each group.^38^ ORs were then converted to SMDs.^39^ If a study reported multiple primary outcomes, their SMDs were averaged to compute a single SMD average per study.

A DerSimonian–Laird random-effects model was used to pool clinical effectiveness data from 53 studies, with a fixed-effects model employed for sensitivity analysis.^38^ Heterogeneity was assessed using the I^2^ statistic. Subgroup analyses were conducted to explore whether clinical effectiveness differed by the presence or absence of five commonly used BCTs, selected based on frequency of use across the included studies.

To further describe patterns in effect sizes, studies were categorized into bins according to Cohen’s conventional thresholds for effect size magnitude (negligible 0–0.20, small 0.20–0.49, medium 0.50–0.79, and large ≥0.80).^40^ To separate positive effects from negative, another bin was created for negative values (negative <0). Frequencies of BCT use were summarized descriptively across these categories. Meta-regression analyses were also conducted to examine the association between effect size and (i) the number of BCTs used in an intervention and (ii) the number of BCT categories represented.

To assess whether the theoretical foundation of an intervention influenced its clinical effectiveness, we conducted a subgroup analysis comparing outcomes across studies categorized by their underlying theory. In this analysis, we examined the broader behavioural theories and models that informed intervention design, while excluding frameworks derived from the BCT taxonomy (e.g., the Behaviour Change Wheel). The analysis focused on established theories commonly applied in health behaviour research, such as Social Cognitive Theory and Self-Determination Theory.^41,42^ All analyses were conducted using STATA 19.^43^

#### Study completion

Study completion was reported as the proportion of participants who completed the follow-up period. For each study arm (intervention and control), the number of participants who completed the study was divided by the total number randomized to that arm. Completion rates were expressed as percentages and summarized descriptively using the mean, standard deviation (SD), median, interquartile range (IQR), and range.

#### Study adherence

No standardized definition of adherence exists for eHealth intervention in older adults.^44^ Therefore, we recorded adherence according to the definitions reported in each study. When multiple adherence definitions were given, the one related to physical activity was used. For each study arm (intervention and control), adherence was calculated as the number of participants who adhered divided by the number randomized to that arm. Adherence was expressed as percentages and summarized descriptively using the mean, standard deviation (SD), median, interquartile range (IQR), and range.

#### Adverse events

When reported, data on the frequency, severity, and type of adverse events were extracted. For studies that provided sufficient information, summary statistics were calculated, including the mean, standard deviation (SD), median, interquartile range (IQR), and range. Safety monitoring procedures were also summarized when described. When intervention groups report adverse events as a binary measure, the frequency of adverse events was recorded as the number of events per participant. The severity of adverse event was coded based on the five-grade (1–5) classification system recommended by the 2004 CONSORT extension for harms, ranging from mild (Grade 1) to death related to the adverse event (Grade 5).^45^

#### Risk of bias and sensitivity analyses

The risk of bias for the primary outcome(s) of each randomized controlled trial (RCT) was assessed using the Cochrane Risk of Bias 2.0 tool (RoB-2).^46^ Risk was categorized as low, some concerns, or high. Two independent reviewers separately assessed all 87 included studies to ensure consistency, as per the Cochrane Handbook for Systematic Reviews of Interventions.^34^ A funnel plot was generated to evaluate the potential for publication bias among studies used in clinical effectiveness analyses (n= 53 studies). If asymmetry was observed, Egger’s test for small-study effects would be done to provide further evaluation. A sensitivity analysis excluding the most influential outlier study was also conducted to assess the robustness of the pooled effect estimate. To assess the potential influence of study quality on intervention effectiveness, subgroup analyses were conducted according to RoB-2 classification (low risk, some concerns, and high risk). Pooled effect sizes were calculated for each risk of bias category, and a test for subgroup differences was done to examine whether effect estimates differed between groups.

## Results

### Study selection

**Figure 1.**
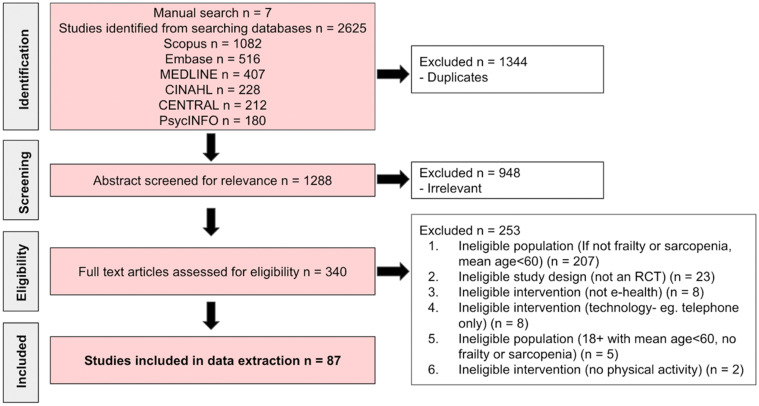
PRISMA 2020 flow diagram of reviewed and included studies.

Following the initial search, 2,625 records were identified (Figure 1). After removing duplicates, 1,284 remained. Title and abstract screening excluded 937 studies, leaving 347 for full-text review. From a hand search of review paper references, an additional 3 studies were added. Of these, 87 RCTs met the inclusion criteria and were included in the analysis. The most common reasons for exclusion were an ineligible study design and a mean participant age below 60 years (without a specific focus on frailty or sarcopenia).

### Study and intervention characteristics

Table 1 shows summary characteristics for each study. Across 86 studies reporting participant age (of 87), the overall mean age was 66.4 years (SD = 5.3), with 66.2 years (SD = 5.1) in intervention groups and 66.1 years (SD = 5.4) in control groups. In total, the 87 studies included 28,988 participants, of whom 52% were female, and 75.3% were white. Education levels varied: 46.3% of participants had completed a baccalaureate degree or higher, whereas 33.6% had completed high school or less. The mean reported BMI was 30.2 kg/m^2^ (SD = 3.6), indicating that most participants were overweight or obese. Although all studies included older adults, many examined additional conditions. The largest subgroup of studies focused exclusively on older adults (n = 27), followed by older adults with cardiovascular disease (n= 18). Only four studies specifically targeted adults with frailty.

**Table 1.** Summary characteristics for each included study (n=87).

Characteristic	Summary
Total number of studies	87 randomized controlled trials
Total participants	28,988 participants
Age, mean (SD)	66.4 (5.3) years overall
	Intervention group: 66.2 (5.1)
	Control group: 66.1 (5.4)
Sex	52.0% female (SD = 28.3)
Race/Ethnicity	75.3% White (SD = 27.4)
Education level	46.3% (SD = 22.4) with ≥ baccalaureate degree
	33.6% (SD = 25.0) with ≤ high school
Body Mass Index	30.2 kg/m^2^ (SD = 3.6)
Health Status	No specified condition: 27 studies (31.0%)
	Cardiovascular: 18 (20.7%)
	Cancer: 14 (16.1%)
	Diabetes: 8 (9.2%)
	Respiratory: 7 (8.0%)
	Musculoskeletal/pain: 7 (8.0%)
	Obesity: 5 (5.7%)
	Frailty: 4 (4.6%);
	Miscellaneous: 4 (4.6%);
	Gastrointestinal: 2 (2.3%)
e-Health Technologies Used	Web-based platform: 43 (49.4%)
	Mobile app: 33 (37.9%)
	Text messages: 25 (28.7%)
	Video calls: 18 (20.7%);
	Email: 9 (10.3%)
Supplementary Devices Used	Wearables: 35 (40.2%)
	Phone calls: 19 (21.8%)
	Other: 11 (12.6%)
Intervention Areas of Focus	PA only: 47 (54.0%);
	PA + Nutrition: 11 (12.6%)
	PA + Nutrition + Other: 10 (11.5%)
	PA + Nutrition + Mental wellness: 9 (10.3%)
	PA + Nutrition + Mental wellness + Other: 5 (5.7%)
	PA + Mental wellness: 4 (4.6%)
	PA + Other: 1 (1.1%)

Most interventions were delivered via web-based platforms (n = 43, 49.4%), followed by mobile applications (n= 33, 37.9%) and text messaging (n= 25, 28.7%). Supplemental components were often used, with 35 studies (40.2%) incorporating wearable devices and 19 (21.8%) telephone support. Control groups most often received usual care, whereas others received minimal interventions, such as educational materials, waitlist controls, or a less advanced version of the intervention. The median intervention duration was 12 weeks, with durations ranging from 2 to 104 weeks.^47,48^ All interventions incorporated a physical activity component. Of these, 47 targeted physical activity alone, 11 targeted both physical activity and nutrition, and 9 focused on physical activity, nutrition, and mental wellness. A detailed table of profile characteristics is provided in Supplemental File 3.^47–133^

### Behaviour change techniques

**Figure 2.**
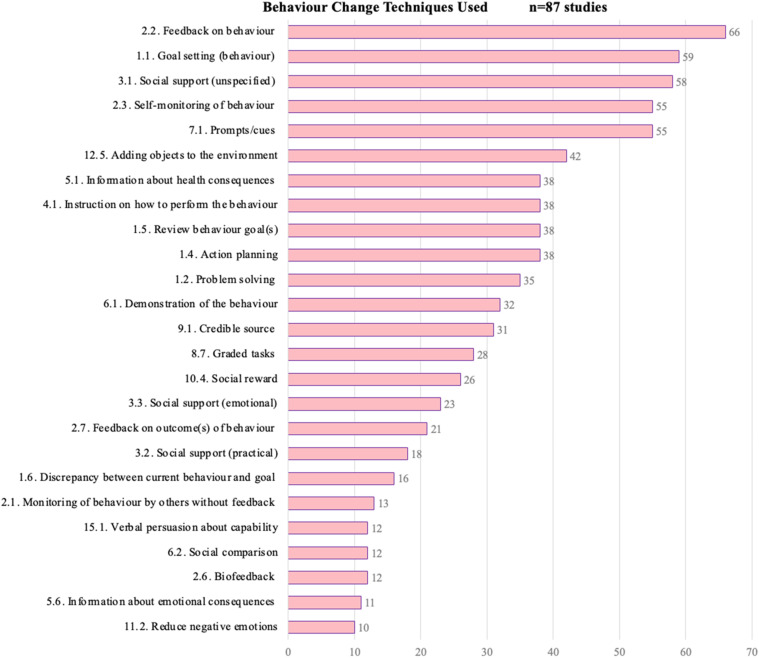
Most commonly used BCTs across interventions (n=87).

The most frequent BCT techniques shared across both intervention and control groups were ‘4.1 Instruction on how to perform the behaviour’ and ‘5.1 Information about health consequences’. Figure 2 presents the frequency of individual BCTs across all 87 interventions. At the individual technique level, the most common were: (i) 2.2 Feedback on behaviour (n = 66 studies), (ii) 1.1 Goal setting (behaviour) (n = 59 studies), and (iii) 3.1 Social support (unspecified) (n = 58 studies). When examined at the broader category level (Table 2), the most frequently applied BCT categories were: (i) Feedback and monitoring (n = 76 studies), (ii) Goals and planning (n = 68 studies), and (iii) Social support (n = 67 studies). Across all 87 studies, the mean number of BCTs used was 10.3 (SD = 5.3), with a median of 10, an IQR of 7 to 13, and a range of 0 to 27. The mean number of BCT categories used per study was 6.4 (SD = 2.6), with a median of 6, an IQR of 5 to 8, and a range from 0 to 13.

**Table 2.** Most commonly used BCT categories across interventions (n=87).

BCT taxonomy categories	Number of interventions to use category	%
Feedback and monitoring	76	87
Goals and Planning	69	79
Social support	67	77
Associations	55	63
Antecedents	47	54
Natural consequences	43	49
Comparison of behaviour	40	46
Shaping knowledge	39	45
Repetition and substitution	34	39
Comparison of outcomes	32	37
Reward and threat	29	33
Self-belief	14	16
Regulation	13	15
Identity	3	3
Scheduled consequences	0	0
Covert learning	0	0

### Clinical effectiveness

Fifty-three studies reported clearly defined primary outcomes (n=91), suitable for meta-analysis. Guided by the COMET outcome framework,^36^ study outcomes were classified into six domains: behavioural (e.g. step count, exercise adherence; n= 37 outcomes), physical functioning and fitness (e.g. VO_2_ max, exercise capacity; n= 17 outcomes), physiological/clinical (e.g. weight change, systolic blood pressure; n= 14 outcomes), psychosocial and quality-of-life (e.g. physical quality of life, self-efficacy for walking; n= 11 outcomes), feasibility and resource-use (e.g. sessions completed, number of days study website visited; n= 10 outcomes), and safety/adverse events (n= 2 outcomes).

**Figure 3.**
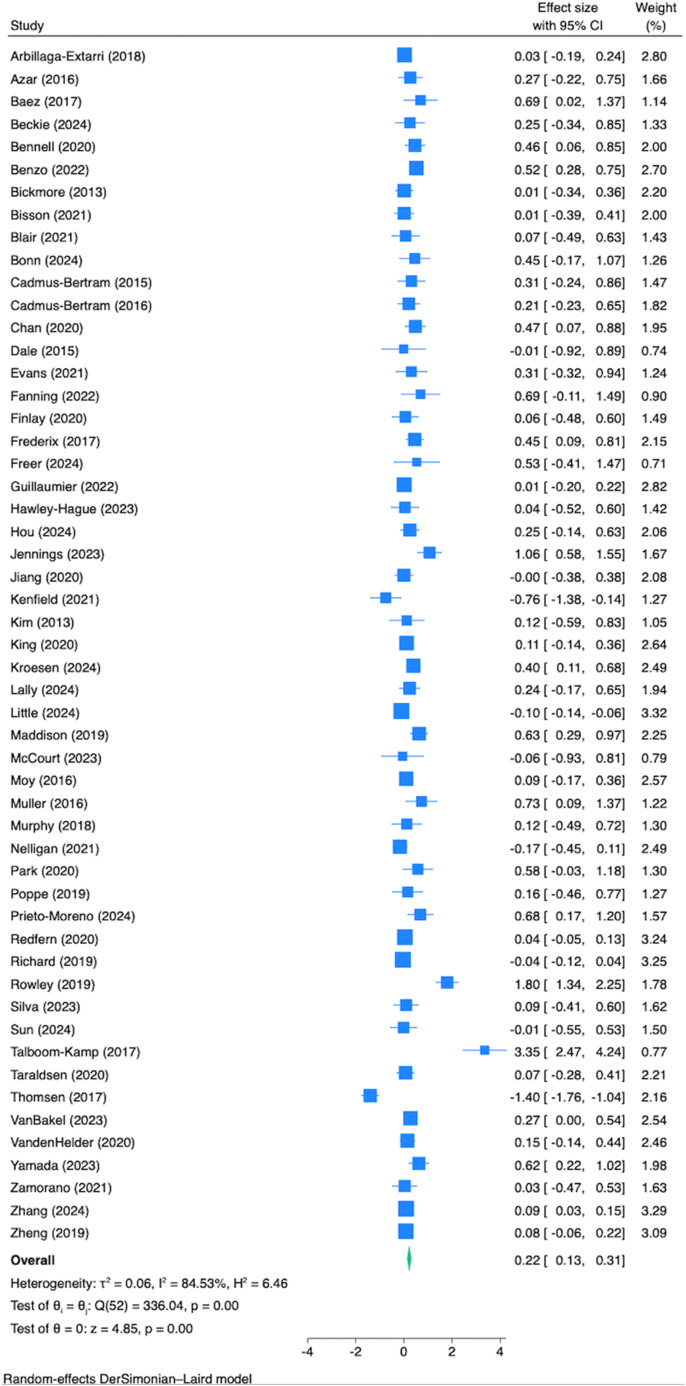
Forest plot of clinical effectiveness across included studies (n= 53).

Outcomes were standardized as effect sizes (SMDs) to enable comparison across heterogeneous measures of clinical effectiveness. The pooled analysis indicated a small but positive effect of interventions compared with controls (SMD = 0.22, 95% CI = 0.13 to 0.31). Heterogeneity was high (I^2^ = 85%), reflecting substantial variability in effect sizes across studies. The forest plot in Figure 3 shows the individual study estimates and the overall pooled effect.

Subgroup analyses were assessed to explore the heterogeneity. Table 3 and Figure 2 show the pooled effect sizes from the subgroup meta-analyses comparing studies with the five BCTs present versus absent. Heterogeneity remained high across all individual subgroups within each of the subgroup analyses (I^2^ > 80%), suggesting that the presence or absence of these five commonly used BCTs did not fully explain the variability observed in the primary meta-analysis. The between-group p-value indicates whether the difference in effect sizes between the two subgroups was statistically significant. For two of the five BCTs used, the observed pooled effect size was larger when the technique was present compared with absent (e.g., BCT ‘2.2 Feedback on behaviour’: 0.27 vs 0.06; BCT ‘3.1 Social support (unspecified)’: 0.30 vs 0.11). A subgroup difference for BCT ‘3.1 Social support (unspecified)’ was non-significant (p=0.05), as were the other subgroup differences (p = 0.13–0.78).

**Table 3.** Subgroup analyses of clinical effectiveness (n= 53 studies) by presence of the five most common BCTs.

BCT	Present: SMD (95% CI)	Absent: SMD (95% CI)	Between-groups p-value
2.2 Feedback on behaviour	0.27 (0.17, 0.37)	0.06 (-0.18, 0.31)	p=0.13
1.1 Goal setting (behaviour)	0.21 (0.09, 0.32)	0.26 (0.10, 0.42)	p=0.59
3.1 Social support (unspecified)	0.30 (0.18, 0.42)	0.11 (-0.04, 0.26)	p=0.05
2.3 Self-monitoring of behaviour	0.22 (0.13, 0.32)	0.26 (0.03, 0.49)	p=0.78
7.1 Prompts and cues	0.21 (0.09, 0.33)	0.24 (0.09, 0.40)	p=0.76

Studies were further grouped by effect size magnitude, and the frequency of BCT use was examined within these categories. Table 4 shows the distribution of studies across the five effect size categories (negative, negligible, small, medium, and large) and the frequency of BCT use within each category. Most studies reported a positive effect size, and the five BCTs showed minimal variation across categories. No statistically significant patterns were observed in BCT use across effect size bins. Although ‘2.2 Feedback on behaviour’ was most common among studies with small positive effects and ‘3.1 Social support (unspecified)’ was among the least common among studies with negative effects, these descriptive findings were not formally tested.

**Table 4.** Distribution of BCTs across effect size bins (present).

BCT	Negative (<0)	Negligible (<0.20)	Small (0.20–0.49)	Medium (0.50–0.79)	Large (≥0.80)
2.2 Feedback on behaviour	77.8%	73.7%	92.3%	66.7%	73.0%
1.1 Goal setting (behaviour)	88.9%	79.0%	69.2%	44.4%	62.2%
3.1 Social support (unspecified)	44.4%	73.7%	69.2%	55.6%	70.3%
2.3 Self-monitoring of behaviour	77.8%	52.6%	76.9%	55.6%	62.2%
7.1 Prompts and cues	66.7%	63.2%	69.2%	66.7%	59.5%

We conducted meta-regressions to explore whether intervention effectiveness was associated with the number of BCTs used or the number of BCT categories. The number of BCTs included in an intervention was not associated with clinical effectiveness (β = 0.002, 95% CI = –0.02 to 0.02, p = 0.86). Meta-regression of BCT categories similarly showed no significant association with clinical effectiveness (β = –0.01, 95% CI = –0.05 to 0.03, p = 0.62). Overall, these analyses did not provide evidence that either the number or type of BCTs systematically influenced clinical effectiveness.

Subgroup analyses were also conducted to assess whether clinical effectiveness differed according to the behavioural theory or model used to inform intervention design (Table 5). Pooled effect sizes were compared between studies in which each theory was present versus absent. The three most applied behavioural theories across studies (n= 53) were Social Cognitive Theory (n = 9), Self-Determination Theory (n = 5), and Self-Efficacy Theory (n = 3). Heterogeneity remained high across all individual subgroups within each of the subgroup analyses (I^2^ > 80%), suggesting that the behavioural theories examined did not fully explain the variability observed in the primary meta-analysis. None of these theories were associated with significantly greater clinical effectiveness (between-groups p = 0.25–0.81).

**Table 5.** Subgroup analyses of clinical effectiveness by presence of the three most common behaviour change theories.

Behavioural theory/Model	Present: SMD (95% CI)	Absent: SMD (95% CI)	Between-groups p-value
Social Cognitive Theory	0.28 (0.03, 0.53)	0.21 (0.11, 0.31)	p=0.63
Self-Determination Theory	0.18 (-0.17, 0.54)	0.23 (0.13, 0.33)	p=0.81
Self-Efficacy Theory	0.40 (0.06, 0.74)	0.20 (0.11, 0.29)	p=0.27

### Study completion

Study completion was reported in 82 studies. In intervention groups, the mean completion rate was 85.1% (SD = 14.8), with a median of 86.9% (IQR = 80.3 to 95.7) and a range of 30.8% to 100%. Control groups showed a similar pattern, with a mean completion rate of 88.0% (SD = 13.0), a median of 91.6% (IQR = 80.4 to 99.6), and a range from 32.4% to 100%. Completion rates varied widely across studies, ranging from 30.8% to 100% in intervention groups and from 32.4% to 100% in control groups.

### Study adherence

Adherence was reported in 32 studies. Definitions included the proportion of prescribed sessions attended, the proportion of participants meeting a predefined usage threshold (e.g., ≥70% of modules completed), or reported mean frequency/duration of use. In intervention groups, the mean adherence rate was 67.8% (SD = 28.0), with a median of 78.4% (IQR = 50 to 92) and a range of 16.2% to 100%. Control groups were either usual care, a waitlist, or a less comprehensive intervention. In control groups that provided a less comprehensive intervention, where adherence was reported, the mean was 46.5% (SD = 26.5), the median was 38.7% (IQR = 30.2 to 64.3), and the range was 10.8% to 87.0%.

### Adverse events

Adverse events were inconsistently reported across 34 studies, with variation in definitions and levels of detail. Due to this inconsistency and missing data, results were synthesized descriptively. Across intervention groups reporting adherence as binary, the mean number of adverse events per participant was 0.15 (SD = 0.40), with a median of 0 (IQR = 0 to 0.13) and a range of 0 to 2.12. In control groups, the mean was 0.14 (SD = 0.40), with a median of 0 (IQR = 0 to 0.06) and a range from 0 to 2.

When adverse event severity was reported, most events in control groups were Grade 1 (mild), accounting for 55 of 58 documented events (94.8%). No moderate events were reported, and three events (5.2%) were classified as Grade 3 serious. Similarly, in the intervention groups, most reported adverse events were mild (118 of 138, 85.5%), with 5 moderate events (3.6%) and 15 serious events (10.9%). No study provided explicit descriptions of safety monitoring procedures beyond researcher oversight.

### Risk of bias and sensitivity analyses

Risk of bias was assessed using the RoB-2 tool.^46^ To ensure consistency, two independent reviewers separately assessed all included studies. Forty studies were rated as having a low risk of bias, twenty-six had some concerns, and twenty-one were considered high risk (Supplemental File 4). Reviewer ratings differed on three studies, and these discrepancies were resolved through discussion with a third reviewer.

Publication bias and sensitivity analyses were conducted to assess the robustness of the findings. Visual inspection of the funnel plot showed large asymmetry (Supplemental File 5), and Egger’s meta-bias test (Supplemental File 6) for small-study effects was significant (p < 0.001). These findings may suggest the presence of publication bias; however, given the substantial heterogeneity across studies (I^2^ = 85%), funnel plot asymmetry may also reflect between-study differences. As such, these findings should be interpreted with caution. A sensitivity analysis excluding the outlier study by Talboom-Kamp et al. produced similar results to the primary meta-analysis (SMD = 0.19, 95% CI 0.11 to 0.27; I^2^ = 82%), indicating that the overall findings were not driven by this study.

Subgroup analyses of the 53 studies included in the meta-analysis were conducted by RoB-2 classification (Supplemental File 7). Studies rated as having a low risk of bias demonstrated a smaller pooled effect size (SMD = 0.13, 95% CI 0.01 to 0.25) than studies with some concerns (SMD = 0.37, 95% CI 0.21 to 0.53) or high risk of bias (SMD = 0.39, 95% CI -0.09 to 0.88). The between-groups difference were not statistically significant (p = 0.05).

## Discussion

This review synthesized 87 RCTs of eHealth interventions delivered to older, frail, or sarcopenic adults, with 53 contributing to our meta-analysis showing a small but positive effect on physical activity (SMD = 0.22, 95% confidence interval: 0.13 to 0.31) despite substantial heterogeneity (I^2^ = 85%). Among the five most common BCTs, none were statistically significant, with the lowest p-value (p=0.05) obtained for ‘social support (unspecified)’. Completion rate (85.1%) and adherence (67.8%) were moderate to high, and although adverse events were rarely reported, they were generally mild, indicating that eHealth interventions can be acceptable and feasible for older adults.

Previous reviews of largely healthy, community-dwelling older adults have also reported small improvements in physical activity with eHealth and other technology-supported interventions, indicating that modest but meaningful gains are possible across populations.^18,134–136^ By incorporating frail populations and systematically coding BCTs, this review extends earlier work^137,138^ and aligns with broader behavior change literature showing that modest effect sizes are common when complex interventions are delivered in real-world.^135,136^

Consistent with previous reviews of eHealth and physical activity, goal setting, monitoring, feedback, and social support remain prominent techniques across interventions.^30,139,140^ In this review, subgroup analyses showed larger effect sizes among studies that included ‘social support (unspecified)’, although the between-group difference was not significant. It remains plausible that social support is an important component for this higher-risk population who may face motivational, functional, and confidence-related barriers, however, this finding should be interpreted cautiously given the non-significant findings, and substantial heterogeneity across studies. Evidence from reviews and meta-analyses in other adult populations reported associations between social support and physical activity outcomes,^25,139,141^ although findings across the literature are mixed. Several syntheses report null or inconsistent associations, and some have even observed negative effects, suggesting that the effectiveness of these common BCTs may depend on how they are combined, delivered, and contextualized.^142,143^

This review also supports longstanding concerns about the variability in how adherence, engagement, and safety are defined and reported in eHealth research. Similar to earlier syntheses, studies varied widely in the metrics, thresholds, and time frames used to measure engagement, which limits our ability to compare interventions or identify dose-response relationships.^144,145^ The sparse and inconsistent reporting of adverse events further reflects patterns seen in digital and exercise trials more broadly, where safety procedures are often implied but not fully documented, highlighting the need for more transparent reporting frameworks.^146^

### Implications for practice, design & research

Collectively, these findings suggest that eHealth interventions can produce small improvements in physical activity and may be feasible for older or frail adults, with moderate-to-high completion and adherence rates where reported. For clinicians and program developers, this supports the continued development of accessible digital interventions for this population, while recognizing that the most effective intervention components remain uncertain. The most commonly used BCTs, including goal setting, monitoring, feedback, and social support, are also reflected in emerging practice models in cardiac rehabilitation, fall prevention, and chronic disease management, suggesting that these approaches may offer useful design considerations for older adults with varying levels of vulnerability.^147–149^

From a research perspective, the review highlights the need for more consistent, theory-informed reporting of intervention content, particularly when BCTs or behavioural frameworks guide the design. Clearer documentation of how intervention components are chosen, operationalized, and delivered would enable more robust testing of hypothesized mechanisms and support cross-trial comparisons of which BCT combinations are most effective in older and frail populations. Standardized definitions and metrics for adherence, engagement, and adverse events are also important for improving comparability and assessing the safety and scalability of eHealth interventions in routine care.^144,150,151^

### Limitations

Several limitations are acknowledged. Despite an extensive search strategy, relevant studies published in languages other than English may have been missed, and some grey literature may not have been captured. The literature search was conducted on October 8, 2024. Studies published after this date were not included and may influence the current evidence base. Substantial heterogeneity across trials, including participant characteristics, intervention components, delivery formats, and control conditions, limited our ability to generate effect estimates, a challenge common to eHealth and complex behavioural interventions.^136,139^ Additionally, the definitions and reporting methods of adherence and adverse events varied considerably across studies, reducing our ability to synthesize these outcomes. As such, these findings should be interpreted carefully. Very few trials recruited people with frailty, while none targeted sarcopenia, restricting applicability to these highest-risk groups. A further limitation relates to the coding of BCTs. To identify intervention-specific components, BCTs present in both intervention and control groups were excluded from all analyses, as their effects could not be attributed specifically to the intervention. However, this coding approach did not capture potential differences in the frequency, intensity, or delivery of BCTs shared across study arms.

Publication bias and study quality should also be considered when interpreting these findings. Funnel plot asymmetry and significant small-study effects suggest the potential presence of publication bias, although these findings may also reflect the substantial heterogeneity observed across studies. In addition, subgroup analyses by RoB-2 classification showed larger effect sizes among studies with some concerns or high risk of bias than among studies with low risk of bias. As such, the pooled effect should be interpreted with caution.

## Conclusions

To our knowledge, this is the largest review to date applying BCT coding to eHealth interventions for older, frail, or sarcopenic adults. It suggests the potential for eHealth approaches to support physical activity among older adults with varying levels of vulnerability. Our findings indicate that eHealth can produce small but positive improvements in physical activity among older adults, with generally acceptable completion rates, moderate adherence, and few reported adverse events. Feedback on behaviour, goal setting, social support, self-monitoring, and prompts/cues were among the most commonly used BCTs across interventions. Overall, these findings support the role of eHealth interventions in fostering healthy aging and maintaining functional independence, while also emphasizing the importance of adapting and testing these approaches for frail and sarcopenic adults who are likely to benefit most.

## Supplemental material

Supplemental material - Behaviour change techniques in eHealth interventions for older, frail, or sarcopenic adults: A systematic review and meta-analysis Supplemental material for Behaviour change techniques in eHealth interventions for older, frail, or sarcopenic adults: A systematic review and meta-analysis by Ayushi Omkar, Jennifer K. Bertrand, Chikku Sadasivan, Emma Osness, Ben Vandermeer, Xiaoxu Ding, Liz Dennett, Julian Mansour, Victor E. Ezeugwu, Ashley Hyde, Puneeta Tandon in DIGITAL HEALTH.

Supplemental material - Behaviour change techniques in eHealth interventions for older, frail, or sarcopenic adults: A systematic review and meta-analysis Supplemental material for Behaviour change techniques in eHealth interventions for older, frail, or sarcopenic adults: A systematic review and meta-analysis by Ayushi Omkar, Jennifer K. Bertrand, Chikku Sadasivan, Emma Osness, Ben Vandermeer, Xiaoxu Ding, Liz Dennett, Julian Mansour, Victor E. Ezeugwu, Ashley Hyde, Puneeta Tandon in DIGITAL HEALTH.

Supplemental material - Behaviour change techniques in eHealth interventions for older, frail, or sarcopenic adults: A systematic review and meta-analysis Supplemental material for Behaviour change techniques in eHealth interventions for older, frail, or sarcopenic adults: A systematic review and meta-analysis by Ayushi Omkar, Jennifer K. Bertrand, Chikku Sadasivan, Emma Osness, Ben Vandermeer, Xiaoxu Ding, Liz Dennett, Julian Mansour, Victor E. Ezeugwu, Ashley Hyde, Puneeta Tandon in DIGITAL HEALTH.

Supplemental material - Behaviour change techniques in eHealth interventions for older, frail, or sarcopenic adults: A systematic review and meta-analysis Supplemental material for Behaviour change techniques in eHealth interventions for older, frail, or sarcopenic adults: A systematic review and meta-analysis by Ayushi Omkar, Jennifer K. Bertrand, Chikku Sadasivan, Emma Osness, Ben Vandermeer, Xiaoxu Ding, Liz Dennett, Julian Mansour, Victor E. Ezeugwu, Ashley Hyde, Puneeta Tandon in DIGITAL HEALTH.

Supplemental material - Behaviour change techniques in eHealth interventions for older, frail, or sarcopenic adults: A systematic review and meta-analysis Supplemental material for Behaviour change techniques in eHealth interventions for older, frail, or sarcopenic adults: A systematic review and meta-analysis by Ayushi Omkar, Jennifer K. Bertrand, Chikku Sadasivan, Emma Osness, Ben Vandermeer, Xiaoxu Ding, Liz Dennett, Julian Mansour, Victor E. Ezeugwu, Ashley Hyde, Puneeta Tandon in DIGITAL HEALTH.

Supplemental material - Behaviour change techniques in eHealth interventions for older, frail, or sarcopenic adults: A systematic review and meta-analysis Supplemental material for Behaviour change techniques in eHealth interventions for older, frail, or sarcopenic adults: A systematic review and meta-analysis by Ayushi Omkar, Jennifer K. Bertrand, Chikku Sadasivan, Emma Osness, Ben Vandermeer, Xiaoxu Ding, Liz Dennett, Julian Mansour, Victor E. Ezeugwu, Ashley Hyde, Puneeta Tandon in DIGITAL HEALTH.

Supplemental material - Behaviour change techniques in eHealth interventions for older, frail, or sarcopenic adults: A systematic review and meta-analysis Supplemental material for Behaviour change techniques in eHealth interventions for older, frail, or sarcopenic adults: A systematic review and meta-analysis by Ayushi Omkar, Jennifer K. Bertrand, Chikku Sadasivan, Emma Osness, Ben Vandermeer, Xiaoxu Ding, Liz Dennett, Julian Mansour, Victor E. Ezeugwu, Ashley Hyde, Puneeta Tandon in DIGITAL HEALTH.

## Data Availability

All data generated or analysed during this study are included in this published article and its supplementary files.*
