# Supplementary material for: Behaviour change techniques in eHealth interventions for older, frail, or sarcopenic adults: A systematic review and meta-analysis
Source: Digit Health. 2026 Jul 28;12:20552076261473804. doi: 10.1177/20552076261473804 (PMC13420075; doi:10.1177/20552076261473804)
Supplement: Supplemental material - Behaviour change techniques in eHealth interventions for older, frail, or sarcopenic adults: A systematic review and meta-analysis [file sj-pdf-2-dhj-10.1177_20552076261473804.pdf]

S2 Text. Detailed search strategy.

Databases: Medline. Embase (no conf abs), Psycinfo, CINAHL, Scopus, Cochrane trials
No language limits
No date limits

Ovid MEDLINE(R) ALL <1946 to October 04, 2024>
Date searched: Oct 8, 2024
Results: 407

1 frailty/ or sarcopenia/ 22605
2 (frail* or sarcopenia).mp. 64205
3 exp aged/ or ("over 65" or "65 and over" or "65 or over" or "65+ years" or frail* or retired or sarcopeni* or elder* or old* old or old age or centenarian* or nonagenarian* or octogenarian* or septuagenarian* or aging or (senior* not ((high school or university or college) adj3 senior*)) or gerontolog* or geriatric* or veteran* or post-menopaus* or postmenopaus* or grandparent* or grandmother* or grandfather* or mature adult* or mature patient* or mature individual* or mature resident* or aged adult* or aged patient* or aged individual* or aged resident* or aged donor* or aged population* or aged care or nursing home resident* or nursing home patient* or (older adj2 (people or person* or client* or resident* or adult* or patient* or individual* or donor* or population* or women or men))).mp. 4250999
4 1 or 2 or 3 4250999
5 Internet-Based Intervention/ 1417
6 ((Online or virtual or web or internet or website* or remote* or video* or app or apps or application* or digital or digiti* or "cellular telephone*" or "mobile telephone*" or "mobile phone*" or "cell phone*" or tele or zoom or skype or google-meet or webex or web-ex or tablet or ipad or "mobile device*" or smartphone* or iphone* or cellphone* or sms or "short messag* service" or "text messag*" or "video conferenc*" or videoconferenc* or teleconferenc* or tele-conferen* or video-to-home or video-visit* or video-technology or tech*-assisted or tech*-supported or tech*-enabled or tech*-based) adj6 (intervention* or program* or initiative* or implement* or model or models or approach or approaches or strategy or strategies or management or self-care or self-manag* or group or groups)).mp. 292337
7 (telehealth or tele-health or "distance health*" or electronic-health* or ehealth or e-health or "digital health").mp. 89910
8 ((remote* or online or virtual* or electronic* or distan* or web or internet) adj2 deliver*).mp. 17262
9 5 or 6 or 7 or 8 381109
10 exercise/ or muscle stretching exercises/ or exp physical conditioning, human/ or swimming/ or walking/ or stair climbing/ or exp Exercise Movement Techniques/ or exp exercise therapy/ or Physical Exertion/ or exp Physical Fitness/ or motor activity/ or locomotion/ or movement/ 534796
11 (exercis* or "physical* activ*" or "motor activity" or "physical* exert*" or fitness or aerobic* or workout* or "strength train*" or "resistance training" or "resistance activities" or "progressive resistance" or "lift weights" or "weight lifting" or weightlifting or "weight training" or "stability training" or "circuit training" or "strengthening program*" or "functional strength" or "functional conditioning" or "home-based strengthening" or "home-based conditioning" or "outpatient strengthening" or "outpatient conditioning" or "progressive strengthening" or "climb stair*" or "stair climb*" or hydrotherap* or aquafit or aquacise or aquasize or aquaaerobic* or calisthenics or danc* or yoga or pilates or "tai chi" or "tai ji" or stretching or stretches or "mobility intervention*" or walking or "regular walk*" or "walk* regularly"

or "postural training" or "postural stability" or mobility or mobilization* or mobilisation* or movement or immobil* or sedentary).mp. 1876216
12 10 or 11 1906973
13 9 and 12 29030
14 ((Online or virtual or web or internet or website* or remote* or video* or app or apps or application* or digital or digiti* or "cellular telephone*" or "mobile telephone*" or "mobile phone*" or "cell phone*" or tele or zoom or skype or google-meet or webex or web-ex or tablet or ipad or "mobile device*" or smartphone* or iphone* or cellphone* or sms or "short messag* service" or "text messag*" or "video conferenc*" or videoconferenc* or teleconferenc* or tele-conferen* or video-to-home or video-visit* or video-technology or tech*-supported or tech*-assisted or tech*-enabled or tech*-based) adj8 (exercis* or "physical* activ*" or "motor activity" or "physical* exert*" or fitness or aerobic* or workout* or "strength train*" or "resistance training" or "resistance activities" or "progressive resistance" or "lift weights" or "weight lifting" or weightlifting or "weight training" or "stability training" or "circuit training" or "strengthening program*" or "functional strength" or "functional conditioning" or "home-based strengthening" or "home-based conditioning" or "outpatient strengthening" or "outpatient conditioning" or "progressive strengthening" or "climb stair*" or "stair climb*" or hydrotherap* or aquafit or aquacise or aquasize or aquaaerobic* or calisthenics or danc* or yoga or pilates or "tai chi" or "tai ji" or stretching or stretches or "mobility intervention*" or walking or "regular walk*" or "walk* regularly" or "postural training" or "postural stability" or mobility or movement or mobilization* or mobilisation* or immobil* or sedentary)).mp. 34033
15 (tele-exercis* or teleexercis*).mp. 72
16 13 or 14 or 15 54508
17 ((behavio* adj3 (intervention* or technique* or strategies or approach*)) or (behavio* adj2 chang*) or BCT or "behavio* taxonomy").mp. 117704
18 exp Clinical trial/ or (randomi* or randomly or (random adj4 (allocat* or distribut* or assign*)) or placebo or trial or groups or subgroups or (phase adj1 ("3" or "2" or "1" or III or II or I))).tw. or rct.ti. 4431526
19 4 and 16 and 17 and 18 407

Embase <1974 to 2024 October 07> (Ovid interface)
Date searched: Oct 8, 2024
Results: 516

1 aged/ or aged hospital patient/ or frail elderly/ or institutionalized elderly/ or very elderly/ or elderly care/ or ("over 65" or "65 and over" or "65 or over" or "65+ years" or frail* or retired or sarcopeni* or elder* or old* old or old age or centenarian* or nonagenarian* or octogenarian* or septuagenarian* or aging or (senior* not ((high school or university or college) adj3 senior*)) or gerontolog* or geriatric* or veteran* or post-menopaus* or postmenopaus* or grandparent* or grandmother* or grandfather* or mature adult* or mature patient* or mature individual* or mature resident* or aged adult* or aged patient* or aged individual* or aged resident* or aged donor* or aged population* or aged care or "nursing home resident*" or "nursing home patient*" or (older adj2 (people or person* or client* or resident* or adult* or patient* or individual* or donor* or population* or women or men))).mp. 5049882
2 web-based intervention/ 3603
3 online support group/ 732
4 ((Online or virtual or web or internet or website* or remote* or video* or app or apps or application* or digital or digiti* or "cellular telephone*" or "mobile telephone*" or "mobile phone*" or "cell phone*" or tele or zoom or skype or google-meet or webex or web-ex or tablet or ipad or "mobile device*" or smartphone* or iphone* or cellphone* or sms or "short messag* service" or "text messag*" or "video conferenc*" or videoconferenc* or teleconferenc* or tele-conferen* or video-to-home or video-

visit* or video-technology or tech*-assisted or tech*-supported or tech*-enabled or tech*-based) adj6 (intervention* or program* or initiative* or implement* or model or models or approach or approaches or strategy or strategies or management or self-care or self-manag* or group or groups)).mp. 364595
5 (telehealth or tele-health or "distance health*" or electronic-health* or ehealth or e-health or "digital health").mp. 118428
6 ((remote* or online or virtual* or electronic* or distan* or web or internet) adj2 deliver*).mp. 11563
7 2 or 3 or 4 or 5 or 6 473012
8 exp leg exercise/ or exp arm exercise/ or exp static exercise/ or exp exercise/ or exp aquatic exercise/ or exp low intensity exercise/ or exp muscle exercise/ or exp aerobic exercise/ or exp stretching exercise/ 466761
9 swimming/ or locomotion/ or physical activity/ 356747
10 walking/ 91703
11 exp kinesiotherapy/ 107637
12 fitness/ 45290
13 motor activity/ 49075
14 body movement/ 16103
15 (exercis* or "physical* activ*" or "motor activity" or "physical* exert*" or fitness or aerobic* or workout* or "strength train*" or "resistance training" or "resistance activities" or "progressive resistance" or "lift weights" or "weight lifting" or weightlifting or "weight training" or "stability training" or "circuit training" or "strengthening program*" or "functional strength" or "functional conditioning" or "home-based strengthening" or "home-based conditioning" or "outpatient strengthening" or "outpatient conditioning" or "progressive strengthening" or "climb stair*" or "stair climb*" or hydrotherap* or aquafit or aquacise or aquasize or aquaaerobic* or calisthenics or danc* or yoga or pilates or "tai chi" or "tai ji" or stretching or stretches or "mobility intervention*" or walking or "regular walk*" or "walk* regularly" or "postural training" or "postural stability" or mobility or mobilization* or mobilisation* or movement or immobil* or sedentary).mp. 2369618
16 or/8-15 2471177
17 7 and 16 42759
18 ((Online or virtual or web or internet or website* or remote* or video* or app or apps or application* or digital or digiti* or "cellular telephone*" or "mobile telephone*" or "mobile phone*" or "cell phone*" or tele or zoom or skype or google-meet or webex or web-ex or tablet or ipad or "mobile device*" or smartphone* or iphone* or cellphone* or sms or "short messag* service" or "text messag*" or "video conferenc*" or videoconferenc* or teleconferenc* or tele-conferen* or video-to-home or video-visit* or video-technology or tech*-supported or tech*-assisted or tech*-enabled or tech*-based) adj8 (exercis* or "physical* activ*" or "motor activity" or "physical* exert*" or fitness or aerobic* or workout* or "strength train*" or "resistance training" or "resistance activities" or "progressive resistance" or "lift weights" or "weight lifting" or weightlifting or "weight training" or "stability training" or "circuit training" or "strengthening program*" or "functional strength" or "functional conditioning" or "home-based strengthening" or "home-based conditioning" or "outpatient strengthening" or "outpatient conditioning" or "progressive strengthening" or "climb stair*" or "stair climb*" or hydrotherap* or aquafit or aquacise or aquasize or aquaaerobic* or calisthenics or danc* or yoga or pilates or "tai chi" or "tai ji" or stretching or stretches or "mobility intervention*" or walking or "regular walk*" or "walk* regularly" or "postural training" or "postural stability" or mobility or movement or mobilization* or mobilisation* or immobil* or sedentary)).mp. 45086
19 (tele-exercis* or teleexercis*).mp. 66
20 17 or 18 or 19 76496
21 ((behavio* adj3 (intervention* or technique* or strategies or approach*)) or (behavio* adj2 chang*) or BCT or "behavio* taxonomy").mp. 172210

22 exp Clinical trial/ or (randomi* or randomly or (random adj4 (allocat* or distribut* or assign*)) or placebo or trial or groups or subgroups or (phase adj1 ("3" or "2" or "1" or III or II or I))).tw. or rct.ti. 6515508
23 1 and 20 and 21 and 22 621
24 limit 23 to conference abstracts 105
25 23 not 24 516

APA PsycInfo <1806 to October 2024 Week 2>(OVID interface)
Date searched: Oct 8, 2024
Results: 180

1 ("380" or "390").ag. or ("over 65" or "65 and over" or "65 or over" or "65+ years" or frail* or retired or sarcopeni* or elder* or old* old or old age or centenarian* or nonagenarian* or octogenarian* or septuagenarian* or aging or (senior* not ((high school or university or college) adj3 senior*)) or gerontolog* or geriatric* or veteran* or post-menopaus* or postmenopaus* or grandparent* or grandmother* or grandfather* or mature adult* or mature patient* or mature individual* or mature resident* or aged adult* or aged patient* or aged individual* or aged resident* or aged donor* or aged population* or aged care or nursing home resident* or nursing home patient* or (older adj2 (people or person* or client* or resident* or adult* or patient* or individual* or donor* or population* or women or men))).mp. 586648
2 online therapy/ or telemedicine/ 14062
3 ((Online or virtual or web or internet or website* or remote* or video* or app or apps or application* or digital or digiti* or "cellular telephone*" or "mobile telephone*" or "mobile phone*" or "cell phone*" or tele or zoom or skype or google-meet or webex or web-ex or tablet or ipad or "mobile device*" or smartphone* or iphone* or cellphone* or sms or "short messag* service" or "text messag*" or "video conferenc*" or videoconferenc* or teleconferenc* or tele-conferen* or video-to-home or video-visit* or video-technology or tech*-assisted or tech*-supported or tech*-enabled or tech*-based) adj6 (intervention* or program* or initiative* or implement* or model or models or approach or approaches or strategy or strategies or management or self-care or self-manag* or group or groups)).mp. 106833
4 (telehealth or tele-health or "distance health*" or electronic-health* or ehealth or e-health or "digital health").mp. 14687
5 ((remote* or online or virtual* or electronic* or distan* or web or internet) adj2 deliver*).mp. 5606
6 2 or 3 or 4 or 5 125791
7 exp exercise/ or physical activity/ or exercise therapy/ or kinesiology/ or movement therapy/ or physical fitness/ or physical endurance/ or physical strength/ 63570
8 (exercis* or "physical* activ*" or "motor activity" or "physical* exert*" or fitness or aerobic* or workout* or "strength train*" or "resistance training" or "resistance activities" or "progressive resistance" or "lift weights" or "weight lifting" or weightlifting or "weight training" or "stability training" or "circuit training" or "strengthening program*" or "functional strength" or "functional conditioning" or "home-based strengthening" or "home-based conditioning" or "outpatient strengthening" or "outpatient conditioning" or "progressive strengthening" or "climb stair*" or "stair climb*" or hydrotherap* or aquafit or aquacise or aquasize or aquaaerobic* or calisthenics or danc* or yoga or pilates or "tai chi" or "tai ji" or stretching or stretches or "mobility intervention*" or walking or "regular walk*" or "walk* regularly" or "postural training" or "postural stability" or mobility or mobilization* or mobilisation* or movement or immobil* or sedentary).mp. 373440
9 7 or 8 374669
10 6 and 9 9409
11 ((Online or virtual or web or internet or website* or remote* or video* or app or apps or application* or digital or digiti* or "cellular telephone*" or "mobile telephone*" or "mobile phone*" or "cell phone*" or tele or zoom or skype or google-meet or webex or web-ex or tablet or ipad or "mobile

device*" or smartphone* or iphone* or cellphone* or sms or "short messag* service" or "text messag*" or "video conferenc*" or videoconferenc* or teleconferenc* or tele-conferen* or video-to-home or video-visit* or video-technology or tech*-supported or tech*-assisted or tech*-enabled or tech*-based) adj8 (exercis* or "physical* activ*" or "motor activity" or "physical* exert*" or fitness or aerobic* or workout* or "strength train*" or "resistance training" or "resistance activities" or "progressive resistance" or "lift weights" or "weight lifting" or weightlifting or "weight training" or "stability training" or "circuit training" or "strengthening program*" or "functional strength" or "functional conditioning" or "home-based strengthening" or "home-based conditioning" or "outpatient strengthening" or "outpatient conditioning" or "progressive strengthening" or "climb stair*" or "stair climb*" or hydrotherap* or aquafit or aquacise or aquasize or aquaaerobic* or calisthenics or danc* or yoga or pilates or "tai chi" or "tai ji" or stretching or stretches or "mobility intervention*" or walking or "regular walk*" or "walk* regularly" or "postural training" or "postural stability" or mobility or movement or mobilization* or mobilisation* or immobil* or sedentary)).mp. 10552

12 (tele-exercis* or teleexercis*).mp. 6

13 10 or 11 or 12 16863

14 ((behavio* adj3 (intervention* or technique* or strategies or approach*)) or (behavio* adj2 chang*) or BCT or "behavio* taxonomy").mp. 106974

15 exp Clinical trials/ or clinical trial.md. or (randomi* or randomly or (random adj4 (allocat* or distribut* or assign*)) or placebo or trial or groups or subgroups or (phase adj1 ("3" or "2" or "1" or III or II or I))).tw. or rct.ti. 863333

16 1 and 13 and 14 and 15 180

CINAHL Plus with Full Text (EBSCOhost interface)
Date searched: Oct 8, 2024
Results: 228

S1 (MH "Aged+") or (frail* or sarcopeni* or "over 65" or "65 and over" or "65 or over" or "65+ years" or retired or elder* or "old* old" or "old age" or centenarian* or nonagenarian* or octogenarian* or septuagenarian* or aging or (senior* NOT (("high school" or university or college) N3 senior*)) or gerontolog* or geriatric* or veteran* or post-menopaus* or postmenopaus* or grandparent* or grandmother* or grandfather* or "mature adult*" or "mature patient*" or "mature individual*" or "mature resident*" or "aged adult*" or "aged patient*" or "aged individual*" or "aged resident*" or "aged donor*" or "aged population*" or "aged care" or "nursing home resident*" or "nursing home patient*" or (older N2 (people or person* or client* or resident* or adult* or patient* or individual* or donor* or population* or women or men)))

S2 ((MH "Internet-Based Intervention") OR ((Online or virtual or web or internet or website* or remote* or video* or app or apps or application* or digital or digiti* or "cellular telephone*" or "mobile telephone*" or "mobile phone*" or "cell phone*" or tele or zoom or skype or google-meet or webex or web-ex or tablet or ipad or "mobile device*" or smartphone* or iphone* or cellphone* or sms or "short messag* service" or "text messag*" or "video conferenc*" or videoconferenc* or teleconferenc* or tele-conferen* or video-to-home or video-visit* or video-technology or tech*-assisted or tech*-supported or tech*-enabled or tech*-based) N6 (intervention* or program* or initiative* or implement* or model or models or approach or approaches or strategy or strategies or management or self-care or self-manag* or group or groups))) OR (telehealth or tele-health or "distance health*" or electronic-health* or ehealth or e-health or "digital health") OR (((remote* or online or virtual* or electronic* or distan* or web or internet) N2 deliver*))

S3 ((MH "Exercise+") OR (MH "Therapeutic Exercise+") OR (MH "Recovery, Exercise") OR (MH "Group Exercise") OR (MH "Aerobic Exercises+") OR (MH "Walking+") OR (MH "Core Exercises") OR (MH "Lower Extremity Exercises") OR (MH "Muscle Strengthening+") OR (MH "Pilates") OR (MH "Stretching") OR (MH "Upper Extremity Exercises+") OR (MH "Physical Activity") OR (MH "Physical Fitness") OR (MH "Cardiorespiratory Fitness") or (MH "Motor Activity") or (MH "Movement") OR (MH "Locomotion") OR (MH "Physical Mobility") OR (MH "Stair Climbing")) OR (exercis* or "physical* activ*" or "motor activity" or "physical* exert*" or fitness or aerobic* or workout* or "strength train*" or "resistance training" or "resistance activities" or "progressive resistance" or "lift weights" or "weight lifting" or weightlifting or "weight training" or "stability training" or "circuit training" or "strengthening program*" or "functional strength" or "functional conditioning" or "home-based strengthening" or "home-based conditioning" or "outpatient strengthening" or "outpatient conditioning" or "progressive strengthening" or "climb stair*" or "stair climb*" or hydrotherap* or aquafit or aquacise or aquasize or aquaaerobic* or calisthenics or danc* or yoga or pilates or "tai chi" or "tai ji" or stretching or stretches or "mobility intervention*" or walking or "regular walk*" or "walk* regularly" or "postural training" or "postural stability" or mobility or mobilization* or mobilisation* or movement or immobil* or sedentary)

S4 S2 AND S3

S5 ((Online or virtual or web or internet or website* or remote* or video* or app or apps or application* or digital or digiti* or "cellular telephone*" or "mobile telephone*" or "mobile phone*" or "cell phone*" or tele or zoom or skype or google-meet or webex or web-ex or tablet or ipad or "mobile device*" or smartphone* or iphone* or cellphone* or sms or "short messag* service" or "text messag*" or "video conferenc*" or videoconferenc* or teleconferenc* or tele-conferen* or video-to-home or video-visit* or video-technology or tech*-supported or tech*-assisted or tech*-enabled or tech*-based) N8 (exercis* or "physical* activ*" or "motor activity" or "physical* exert*" or fitness or aerobic* or workout* or "strength train*" or "resistance training" or "resistance activities" or "progressive resistance" or "lift weights" or "weight lifting" or weightlifting or "weight training" or "stability training" or "circuit training" or "strengthening program*" or "functional strength" or "functional conditioning" or "home-based strengthening" or "home-based conditioning" or "outpatient strengthening" or "outpatient conditioning" or "progressive strengthening" or "climb stair*" or "stair climb*" or hydrotherap* or aquafit or aquacise or aquasize or aquaaerobic* or calisthenics or danc* or yoga or pilates or "tai chi" or "tai ji" or stretching or stretches or "mobility intervention*" or walking or "regular walk*" or "walk* regularly" or "postural training" or "postural stability" or mobility or movement or mobilization* or mobilisation* or immobil* or sedentary))

S6 tele-exercis* or teleexercis*

S7 S4 OR S5 OR S6

S8 ((behavio* N3 (intervention* or technique* or strategies or approach*)) or (behavio* N2 chang*) or BCT or "behavio* taxonomy")

S9 ((MH "Clinical Trials+") OR (MH "Community Trials") or randomi* or "randomly" or ("random" N4 (allocat* or distribut* or assign*)) or "placebo" or "trial" or "groups" or "subgroups" OR or (phase N1 ("3" or "2" or "1" or III or II or I)) OR TI(RCT))

S9 S1 AND S7 AND S8 AND S9

Cochrane Library (Trials database only)
Date searched: Oct 8, 2024
Results: 212

#1 ("over 65" or "65 and over" or "65 or over" or "65+ years" or frail* or retired or sarcopeni* or elder* or "oldest old" or "old age" or centenarian* or nonagenarian* or octogenarian* or septuagenarian* or aging or (senior* not (("high school" or university or college) NEAR/3 senior*)) or gerontolog* or geriatric* or veteran* or post-menopausal or postmenopaus* or grandparent* or grandmother* or grandfather* or (mature NEXT (adult* or patient* or individual* or resident*)) or (aged NEXT (adult* or patient* or individual* or resident* or donor* or population* or care)) or (nursing-home NEXT (resident* OR patient*)) or (older NEAR/2 (people or person* or client* or resident* or adult* or patient* or individual* or donor* or population* or women or men))):ti,ab,kw
#2 [mh "aged"] or [mh "frailty"] or [mh "sarcopenia"]
#3 #1 OR #2
#4 [mh ^"Internet-Based Intervention"]
#5 ((Online or virtual or web or internet or website* or remote* or video* or app or apps or application* or digital or digiti* or tele or zoom or skype or google-meet or webex or web-ex or tablet or ipad or ((mobile or cell*) NEXT (device* or phone* or telephone*)) or smartphone* or iphone* or cellphone* or sms or "short message service" or (text NEXT messag*) or (video NEXT conferenc*) or videoconferenc* or teleconferenc* or (tele NEXT conferen*) or video-to-home or (video NEXT visit*) or video-technology or (tech* NEXT (assisted or supported or enabled or based))) NEAR/6 (intervention* or program* or initiative* or implement* or model or models or approach or approaches or strategy or strategies or management or self-care or (self NEXT manag*) or group or groups)):ti,ab,kw
#6 (telehealth or tele-health or "distance health" or electronic-health or ehealth or e-health or "digital health"):ti,ab,kw
#7 ((remote* or online or virtual* or electronic* or distan* or web or internet) NEAR/2 deliver*):ti,ab,kw
#8 #4 OR #5 OR #6 OR #7
#9 [mh ^"exercise"] or [mh ^"muscle stretching exercises"] or [mh "physical conditioning, human"] or [mh ^"swimming"] or [mh ^"walking"] or [mh ^"stair climbing"] or [mh "Exercise Movement Techniques"] or [mh "exercise therapy"] or [mh ^"Physical Exertion"] or [mh "Physical Fitness"] or [mh ^"motor activity"] or [mh ^"locomotion"] or [mh ^"movement"] or (exercis* or (physical* NEXT activ*) or "motor activity" or (physical* NEXT exert*) or fitness or aerobic* or workout* or (strength NEXT train*) or "resistance training" or "resistance activities" or "progressive resistance" or "lift weights" or "weight lifting" or weightlifting or "weight training" or "stability training" or "circuit training" or (strengthening NEXT program*) or "functional strength" or "functional conditioning" or "home-based strengthening" or "home-based conditioning" or "outpatient strengthening" or "outpatient conditioning" or "progressive strengthening" or (climb NEXT stair*) or (stair NEXT climb*) or hydrotherap* or aquafit or aquacise or aquasize or aquaaerobic* or calisthenics or danc* or yoga or pilates or "tai chi" or "tai ji" or stretching or stretches or (mobility NEXT intervention*) or walking or (regular NEXT walk*) or (walk* NEXT regularly) or "postural training" or "postural stability" or mobility or mobilization* or mobilisation* or movement or immobil* or sedentary):ti,ab,kw
#10 #8 AND #9
#11 ((Online or virtual or web or internet or website* or remote* or video* or app or apps or application* or digital or digiti* or tele or zoom or skype or google-meet or webex or web-ex or tablet or ipad or ((mobile or cell*) NEXT (device* or phone* or telephone*)) or smartphone* or iphone* or cellphone* or sms or "short message service" or (text NEXT messag*) or (video NEXT conferenc*) or videoconferenc* or teleconferenc* or (tele NEXT conferen*) or video-to-home or (video NEXT visit*) or video-technology or (tech* NEXT (assisted or supported or enabled or based))) NEAR/8 (exercis* or (physical* NEXT activ*) or "motor activity" or (physical* NEXT exert*) or fitness or aerobic* or workout* or (strength NEXT train*) or "resistance training" or "resistance activities" or "progressive

resistance" or "lift weights" or "weight lifting" or weightlifting or "weight training" or "stability training" or "circuit training" or (strengthening NEXT program*) or "functional strength" or "functional conditioning" or "home-based strengthening" or "home-based conditioning" or "outpatient strengthening" or "outpatient conditioning" or "progressive strengthening" or (climb NEXT stair*) or (stair NEXT climb*) or hydrotherap* or aquafit or aquacise or aquasize or aquaaerobic* or calisthenics or danc* or yoga or pilates or "tai chi" or "tai ji" or stretching or stretches or (mobility NEXT intervention*) or walking or (regular NEXT walk*) or (walk* NEXT regularly) or "postural training" or "postural stability" or mobility or mobilization* or mobilisation* or movement or immobil* or sedentary)):ti,ab,kw
#12 (tele-exercis* or teleexercis*):ti,ab,kw
#13 #10 OR #11 OR #12
#14 ((behavio* NEAR/3 (intervention* or technique* or strategies or approach*)) or (behavio* NEAR/2 chang*) or BCT or (behavio* NEXT taxonomy)):ti,ab,kw
#15 #2 AND #13 AND #14

Scopus (Advanced search)
Date searched: Oct 8 2024
Results: 1082

(KEY (aged) OR TITLE-ABS-KEY (frail* OR sarcopeni* OR "over 65" OR "65 and over" OR "65 or over" OR "65+ years" OR retired OR elder* OR "old* old" OR "old age" OR centenarian* OR nonagenarian* OR octogenarian* OR septuagenarian* OR aging OR (senior* AND NOT (("high school" OR university OR college) W/3 senior*)) OR gerontolog* OR geriatric* OR veteran* OR post-menopaus* OR postmenopaus* OR grandparent* OR grandmother* OR grandfather* OR "mature adult*" OR "mature patient*" OR "mature individual*" OR "mature resident*" OR "aged adult*" OR "aged patient*" OR "aged individual*" OR "aged resident*" OR "aged donor*" OR "aged population*" OR "aged care" OR "nursing home resident*" OR "nursing home patient*" OR (older W/2 (people OR person* OR client* OR resident* OR adult* OR patient* OR individual* OR donor* OR population* OR women OR men))))

AND
(TITLE-ABS-KEY ((((online OR virtual OR web OR internet OR website* OR remote* OR video* OR app OR apps OR application* OR digital OR digiti* OR "mobile telephone*" OR "cell* telephone*" OR "mobile phone*" OR "cell phone*" OR tele OR zoom OR skype OR google-meet OR webex OR web-ex OR tablet OR ipad OR "mobile device*" OR smartphone* OR iphone* OR cellphone* OR sms OR "short messag* service" OR "text messag*" OR "video conferenc*" OR videoconferenc* OR teleconferenc* OR tele-conferen* OR video-to-home OR video-visit* OR video-technology OR tech*-assisted OR tech*-supported OR tech*-enabled OR tech*-based) W/6 (intervention* OR program* OR initiative* OR implement* OR model OR models OR approach OR approaches OR strategy OR strategies OR management OR self-care OR self-manag* OR group OR groups)) OR telehealth OR tele-health OR "distance health*" OR electronic-health* OR ehealth OR e-health OR "digital health" OR ((remote* OR online OR virtual* OR electronic* OR distan* OR web OR internet) W/2 deliver*)) AND (exercis* OR "physical* activ*" OR "motor activity" OR "physical* exert*" OR fitness OR aerobic* OR workout* OR "strength train*" OR "resistance training" OR "resistance activities" OR "progressive resistance" OR "lift weights" OR "weight lifting" OR weightlifting OR "weight training" OR "stability training" OR "circuit training" OR "strengthening program*" OR "functional strength" OR "functional conditioning" OR "home-based strengthening" OR "home-based conditioning" OR "outpatient strengthening" OR "outpatient conditioning" OR "progressive strengthening" OR "climb stair*" OR "stair climb*" OR hydrotherap* OR aquafit OR aquacise OR aquasize OR aquaaerobic* OR calisthenics OR

danc* OR yoga OR pilates OR "tai chi" OR "tai ji" OR stretching OR stretches OR walking OR "regular walk*" OR "walk* regularly" OR "postural training" OR "postural stability" OR mobility OR mobilization* OR mobilisation* OR movement OR immobil* OR sedentary)) OR TITLE-ABS-KEY (((online OR virtual OR web OR internet OR website* OR remote* OR video* OR app OR apps OR application* OR digital OR digiti* OR "mobile telephone*" OR "cell* telephone*" OR "mobile phone*" OR "cell phone*" OR tele OR zoom OR skype OR google-meet OR webex OR web-ex OR tablet OR ipad OR "mobile device*" OR smartphone* OR iphone* OR cellphone* OR sms OR "short messag* service" OR "text messag*" OR "video conferenc*" OR videoconferenc* OR teleconferenc* OR tele-conferen* OR video-to-home OR video-visit* OR video-technology OR tech*-assisted OR tech*-supported OR tech*-enabled) W/8 (exercis* OR "physical* activ*" OR "physical* exert*" OR fitness OR aerobic* OR workout* OR "strength train*" OR "resistance training" OR "resistance activities" OR "progressive resistance" OR "lift weights" OR "weight lifting" OR weightlifting OR "weight training" OR "stability training" OR "circuit training" OR "strengthening program*" OR "functional strength" OR "functional conditioning" OR "home-based strengthening" OR "home-based conditioning" OR "outpatient strengthening" OR "outpatient conditioning" OR "progressive strengthening" OR "climb stair*" OR "stair climb*" OR hydrotherap* OR aquafit OR aquacise OR aquasize OR aquaaerobic* OR calisthenics OR danc* OR yoga OR pilates OR "tai chi" OR "tai ji" OR stretching OR stretches OR "mobility intervention*" OR walking OR "regular walk*" OR "walk* regularly" OR "postural training" OR "postural stability" OR mobility OR movement OR mobilization* OR mobilisation* OR immobil* OR sedentary)) OR tele-exercis* OR teleexercis*))

AND TITLE-ABS-KEY ((behavio* W/3 (intervention* OR technique* OR strategies OR approach*)) OR (behavio* W/2 chang*) OR "behavio* taxonomy") AND (TITLE-ABS-KEY ({Clinical-trial} OR {controlled-trial} OR randomi* OR {randomly} OR (random W/4 (allocat* OR distribut* OR assign*)) OR {placebo} OR {trial} OR {groups} OR {subgroups} OR (phase W/1 ("3" OR "2" OR "1" OR iii OR ii OR i))) OR TITLE (rct))
