# Supplementary material for: Behaviour change techniques in eHealth interventions for older, frail, or sarcopenic adults: A systematic review and meta-analysis
Source: Digit Health. 2026 Jul 28;12:20552076261473804. doi: 10.1177/20552076261473804 (PMC13420075; doi:10.1177/20552076261473804)
Supplement: Supplemental material - Behaviour change techniques in eHealth interventions for older, frail, or sarcopenic adults: A systematic review and meta-analysis [file sj-pdf-3-dhj-10.1177_20552076261473804.pdf]

S3 Table. Detailed characteristic profiles for each included study (n=87).

Author	Year	Sample size (n)	Mage (SD)	Sex F (n%)	Health status	eHealth delivery modality (and supplemental components)	Targeted behavioural domain	Intervention duration	Primary outcome(s)
**Arbillaga-Etxarri [49]**	2018	I: 202<br>C: 205	I: 68 (9)<br>C: 69 (8)	13	Older adults with Chronic obstructive pulmonary disease (COPD)	Web-based platform, wearable device, text messages, phone calls	Physical activity	52 weeks	1. Steps per day
**Azar [50]**	2016	I: 37<br>C: 37	I: 59.6 (11.9)<br>C: 59.8 (10.5)	59.5	Older adults at high risk for Type II Diabetes and/or Cardiovascular disease (CVD)	Web-based platform, mobile app, wearable device, video calls, Other (wireless scale)	Physical activity, nutrition, mental wellness	12 weeks[1]	1. Overall health related quality of life (Short Form-8 Survey Mental)<br>2. Overall health related quality of life (Short Form-8 Survey Physical)
**Baez [51]**	2017	I: 20<br>C: 20	I: 70.3 (4.5)<br>C: 71.5 (6.8)	72.5	Non-frail, transitionally frail, or mildly frail older adults	Web-based platform, mobile app	Physical activity	8 weeks	1. Adherence (persistence rate- %)<br>2. Adherence (completeness rate- %)<br>3. Leg strength: 30 second chair stand<br>4. Mobility: Timed up and go
**Bailey [52]**	2024	I: 28<br>C: 30	I: 75 (7)<br>C: 74 (6)	67	Older adults with very mild or mild frailty	Mobile app, wearable device, text messages, phone calls, video calls, Other (Microsoft teams)	Physical activity	24 weeks	Study completion rate as measured by:<br>1. activPAL data: n(%) valid for ≥4 days<br>2. Sarcopenia: Muscle mass, n(%)<br>3. Sarcopenia: Hand grip strength, n(%)<br>4. Sarcopenia: Short Physical Performance Battery (SPPB), n(%)
**Beckie [53]**	2024	I: 28<br>C: 30	I: 62.7 (7.3)<br>C: 59.7 (10.5)	100	Older adults with Coronary heart disease (CHD)	mobile app, wearable device, text messages, phone calls	Physical activity, nutrition, mental wellness	12 weeks	1. Exercise capacity (6 minute walk distance (6MWD), metres)
**Bennell [54]**	2020	I: 56<br>C: 54	I: 61.7 (6.7)<br>C: 62.9 (6.8)	67.3	Older adults with knee osteoarthritis and high BMI	Text messages	Physical activity	24 weeks	1. Adherence to prescribed home exercise (Exercise Adherence Rating Scale- EARS)<br>2. Number of days home exercise completed in the past week

**Bentley [55]**	2020	I: 19<br>C: 11	I: 68 (6.7)<br>C: 66 (7.4)	56.7	Older adults with COPD	Mobile app, wearable device	Physical activity	6 weeks	NR
**Benzo [56]**	2022	I: 188<br>C: 187	I: 69.3 (9.5)<br>C: 68.7 (9.5)	56.5	Older adults with COPD	Web-based platform, wearable device, phone calls, Other (oximeter)	Physical activity, mental wellness	12 weeks	1. Physical Quality of Life (chronic respiratory questionnaire summary scores)<br>2. Emotional Quality of Life (chronic respiratory questionnaire summary scores)
**Bickmore [57]**	2013	I: 132<br>C: 131	I: 71.7 (5.6)<br>C: 70.8 (5.2)	61.2	Older adults	Web-based platform, wearable device	Physical activity	52 weeks	1. Average daily step count (for 30 days before end of study)
**Bisson [58]**	2021	I: 50<br>C: 46	I: 61.6 (7.7)<br>C: 61.5 (8.1)	70	Older adults	Mobile app, Other (iPhone accelerometer)	Physical activity	4 weeks	1. Average daily step count
**Blair [59]**	2021	I: 36<br>C: 18	I: 69.4 (4.2)<br>C: 70.2 (5.9)	56	Older cancer survivors	Mobile app, wearable device, text messages, phone calls	Physical activity	13 weeks	1. Changes in total sedentary time (average minutes per day)<br>2. Number of breaks from sitting (average breaks per day)
**Bonn [60]**	2024	I: 93<br>C: 88	NR	34.3	Older adults with Type II Diabetes	Mobile app phone calls	Physical activity	12 weeks	1. Moderate to vigorous physical activity (MVPA-mins/day) at 3 months follow-up.
**Bowen [61]**	2022	I: 15<br>C: 15	I: 65.3 (3.5)<br>C: 64.7 (2.9)	100	Older adults with BMI ≥25	Text messages	Physical activity	12 weeks	NR
**Bronas [62]**	2024	I: 20<br>C: 19	I: 59.9 (5.0)<br>C: 62.2 (6.6)	66.7	Older adults	Wearable device, text messages, phone calls, video calls	Physical activity	6 weeks	1. Feasibility<br>2. Acceptability
**Cadmus-Bertram [63]**	2015	I: 25<br>C: 26	I: 58.6 (6.5)<br>C: 61.3 (7.5)	100	Postmenopausal older adults with BMI ≥25	Web-based platform, wearable device, phone calls	Physical activity	16 weeks	1. Total MVPA (minutes/week)<br>2. Average steps per day

**Cadmus-Bertram [64]**	2016	I: 71<br>C: 34	I: 60 (6.3)<br>C: 60.8 (6.2)	100	Older adults with a history of ductal or lobular carcinoma in situ or a Gail model score of 1.7.	Web-based platform, wearable device, phone calls	Physical activity, nutrition, mental wellness	52 weeks	1. Weight change (kg)
**Chan [65]**	2020	I: 153<br>C: 49	I: 70 (7.6)<br>C: 70 (8.9)	0	Older adults with prostate cancer	Web-based platform, wearable device, text messages, phone calls	Physical activity, nutrition	12 weeks	1. Feasibility<br>2. Acceptability
**Dale [66]**	2015	I: 61<br>C: 62	I: 59 (10.5)<br>C: 59.9 (11.8)	18.7	Older adults with CHD	Web-based platform, wearable device, text messages	Physical activity, nutrition, other (smoking cessation)	24 weeks	1. Patient adherence (Nonsmoker)<br>2.Patient adherence (Non-harmful alcohol intake)<br>3. Patient adherence (Physically active)<br>4. Patient adherence (≥5 Fruit and vegetable intake)
**Engelen [67]**	2020	I: 103<br>C: 105	I: 63.3 (10)<br>C: 63.7 (9.8)	31.7	Older adults with CVD	Web-based platform	Physical activity, nutrition, mental wellness	52 weeks	NR
**Evans [68]**	2021	I: 20<br>C: 20	I: 69.5 (6.6)<br>C: 70.8 (10.2)	0	Older adults with metastatic prostate cancer	Web-based platform, text messages, phone calls, video calls	Physical activity, mental wellness	8 weeks	1. Efficacy: Physical activity (MVPA-minutes/day)<br>2. Efficacy: Sedentary activity (minutes/day)<br>3. Efficacy: Steps (steps/day)<br>4. Adverse events
**Fanning [69]**	2020	I: 15<br>C: 13	I: 70.2 (5.4)<br>C: 70.3 (5.2)	78.6	Older adults with chronic multisite pain	Mobile app, wearable device, phone calls, video calls, other (body-trace weight scale)	Physical activity, nutrition, mental wellness	12 weeks	NR
**Fanning [70]**	2022	I: 15<br>C: 13	I: 70.1 (5.4)<br>C: 70.3 (5.2)	78.6	Obese older adults with chronic pain	Mobile app, wearable device, video calls, other (body-trace weight scale)	Physical activity, nutrition, mental wellness	12 weeks	1. Self-efficacy for walking<br>2. Satisfaction with walking<br>3. Health related quality of life for physical functioning (36-item short form survey)
**Fanning [71]**	2023	NR	NR	57.1	Older adults with early-stage Alzheimer's disease participating with a caregiver.	Video calls	Physical activity	12 weeks	NR

**Finlay [72]**	2020	I: 60<br>C: 18	I: 66.9 (9.6)<br>C: 66.8 (9.6)	0	Older adults with a current or previous diagnosis of prostate cancer	Web-based platform, other (emails)	Physical activity	4 weeks	1. Physical activity log engagement
**Frederix [73]**	2017	I: 70<br>C: 70	I: 61 (9)<br>C: 61 (8)	18.3	Older adults with Coronary Artery Disease (CAD), Chronic Heart Failure (CHF), and/or respiratory disease	Web-based platform, wearable device (IMU) (eg. smartwatch), text messages, other (emails)	Physical activity, nutrition, other (smoking cessation, comorbidities)	24 weeks	1. VO2 peak (ml/min*kg)
**Freer [74]**	2024	I: 14<br>C: 14	I: 70.1 (5.4)<br>C: 66.8 (9.6)	92.9	Older adults with self-reported NAFLD	Mobile app, text messages, phone calls, video calls (eg. zoom)	Physical activity	12 weeks	1. Exercise adherence (avg of 3x weekly muscle strengthening program)<br>2. Dietary Adherence (adhered to at least 80% of recommended servings of total protein- n%)<br>3. Dietary Adherence (adhered to at least 80% of recommended servings of total protein- servings/day)
**Gerber [75]**	2024	I: 10<br>C: 10	I: 65.5 (4.6)<br>C: 65.3 (6.3)	45	Older adults	Web-based platform, mobile app, wearable device, video calls, other (Wi-Fi body scale)	Physical activity, nutrition	24 weeks	1. Change in body mass index (BMI)
**Guillaumier [76]**	2022	I: 199<br>C: 200	I: 67 (12)<br>C: 68 (12)	34.8	Stroke survivors	Web-based platform (eg. website with modules or health coach); Text messages	Physical activity; Nutrition ; Mental wellness ; Other: Blood pressure, smoking	12 weeks	1. Health-related quality of life (EuroQol Visual Analogue Scale, EQ-VAS)
**Hawley-Hague [77]**	2023	I: 26<br>C: 24	I: 77 (8.5)<br>C: 78.2 (7.4)	68	Older adults at risk of falls	Mobile app ; Text messages	Physical activity	24 weeks	1. Study Adherence (EARS)
**Hou [78]**	2024	I: 55<br>C: 55	I: 64.5 (0.6)<br>C: 69.2 (0.7)	45.7	Patient with Type II Diabetes	Text messages	Physical activity; Nutrition ; Mental wellness	24 weeks	1. Reductions in hemoglobin A1c (HbA1c) at 6 months<br>2. Fasting blood glucose at 6 months
**Ilie [79]**	2023	I: 72<br>C: 68	I: 66 (7.4)<br>C: 68 (8.1)	0	Patients undergoing curative prostate cancer treatment	Text messages ; Phone calls ; Video calls (eg. zoom); Other: Email, Stress reduction biofeedback device	Physical activity; Nutrition ; Mental wellness ; Other: Communication, sex education, relationships	24 weeks	1. Nonspecific psychological distress

**Jennings [80]**	2024	I: 70<br>C: 34	I: 67.7 (4.7)<br>C: 67.1 (4.4)	74	Older adults at risk of dementia	Web-based platform (eg. website with modules or health coach); Video calls (eg. zoom)	Physical activity; Nutrition	24 weeks	1. Dietary patterns (mediterranean diet adherence screener, MEDAS)<br>2. Physical activity behaviour change
**Jiang [81]**	2020	I: 53<br>C: 53	I: 70.9 (6.4)<br>C: 71.8 (7.6)	18	Patients with COPD	Mobile app	Physical activity; Nutrition ; Other: Medications	12 weeks	1. Symptoms and effects of COPD (chronic obstructive pulmonary disease assessment test, CAT)
**Kenfield [82]**	2019	I: 37<br>C: 39	I: 66 (5.2)<br>C: 65 (6.7)	0	Patients with clinical stage T1-T3a prostate cancer	Web-based platform (eg. website with modules or health coach); Wearable device (IMU) (eg. smartwatch); Text messages ; Other: Email	Physical activity; Nutrition ; Other: Smoking	12 weeks	1. Feasibility<br>2. Acceptability
**Kenfield [83]**	2021	I: 18<br>C: 10	I: 71.4 (4.7)<br>C: 72 (9.6)	0	Patients with prostate cancer	Mobile app ; Wearable device (IMU) (eg. smartwatch); Phone calls ; Other: Email	Physical activity	12 weeks	1. Attendance (attended 70% of more of exercise sessions)<br>2. Adherence to exercise prescription (Number who completed ≥70% exercise sessions as or more than prescribed)<br>3. Tolerance (Median sessional tolerance)<br>4. Adverse events (elsewhere)
**Kim [84]**	2013	I: 30<br>C: 15	I: 69.3 (7.3)<br>C: 70.6 (7.5)	80.6	Older adults	Wearable device (IMU) (eg. smartwatch); Text messages	Physical activity	6 weeks	1. Step count (steps/day)
**King [85]**	2020	I: 123<br>C: 122	I: 63.1 (8.3)<br>C: 62.4 (8.5)	78.8	Inactive older adults	Web-based platform (eg. website with modules or health coach); Wearable device (IMU) (eg. smartwatch)	Physical activity	52 weeks	1. Change in 12-month walking minutes per week
**Kroesen [86]**	2024	I: 112<br>C: 108	I: 63 (10)<br>C: 64 (10)	23	Patients with CAD enrolled in cardiac rehab (CR)	Mobile app ; Wearable device (IMU) (eg. smartwatch)	Physical activity	12 weeks	1. Change in accelerometer-derived sedentary time from pre-CR to post-CR (hours/day)
**Kwan [87]**	2020	I: 16<br>C: 17	I: 70.5 (5.2)<br>C: 71.0 (10.4)	85	Older adults	Mobile app	Physical activity	12 weeks	1. Cognitive function<br>2. Frailty<br>3. Walking time<br>4. MVPA

**Lally [88]**	2024	I: 44<br>C: 46	I: 63 (NR)<br>C: 62 (NR)	48	Adults living with and beyond a cancer diagnosis	Mobile app ; Wearable device (IMU) (eg. smartwatch); Phone calls ; Other: Walking planner	Physical activity	12 weeks	1. Physical activity (ActivPAL data)
**Langlais [89]**	2021	I: 153<br>C: 49	NR	0	Men with prostate cancer	Web-based platform (eg. website with modules or health coach); Wearable device (IMU) (eg. smartwatch); Text messages ; Phone calls	Physical activity; Nutrition	12 weeks	NR
**Laslovich [90]**	2020	I: 19<br>C: 19	I: 68 (7.5)<br>C: 68 (10.6)	55.3	Adults with peripheral artery disease	Web-based platform (eg. website with modules or health coach); Wearable device (IMU) (eg. smartwatch)	Physical activity	12 weeks	NR
**Lindsay [91]**	2009	I: 54<br>C: 54	NR	33.6	Patients with coronary heart disease (CHD)	Web-based platform (eg. website with modules or health coach)	Physical activity; Nutrition	36 weeks	NR
**Little [92]**	2024	I: 10348<br>C: 3451	I: 61.5 (14.4)<br>C: 61.6 (14.4)	54.2	Adults (aged ≥18 years) had at least one comorbidity or risk factor increasing their risk of adverse outcomes due to respiratory illness	Web-based platform (eg. website with modules or health coach); Wearable device (IMU) (eg. smartwatch)	Physical activity	24 weeks	1. Total number of days of illness due to self-reported respiratory tract illnesses (coughs, colds, sore throat, sinus or ear infections, influenza, or COVID-19) in the previous 6 months.
**Lyons [93]**	2017	I: 20<br>C: 20	I: 61.3 (5)<br>C: 61.7 (6.3)	85	Adults with BMI from 25-35	Mobile app ; Wearable device (IMU) (eg. smartwatch)	Physical activity	12 weeks	NR
**Maddison [94]**	2019	I: 82<br>C: 80	I: 61 (13.2)<br>C: 61.5 (12.2)	14.2	Adults with coronary heart disease (CHD)	Web-based platform (eg. website with modules or health coach); Mobile app ; Wearable device (IMU) (eg. smartwatch)	Physical activity	12 weeks	1. VO2max (ml/min*kg)
**McCourt [95]**	2023	I: 23<br>C: 27	I: 59.3 (9.4)<br>C: 61.3 (8.7)	33	Patients with myeloma	Wearable device (IMU) (eg. smartwatch); Video calls (eg. zoom); Other: HR monitor	Physical activity	12 weeks	1. Feasibility

**Mouton [96]**	2015	I: 156<br>C: 50	I: 65.0 (7.5)<br>C: 66.1 (6.8)	62.4	Adults over 50	Web-based platform (eg. website with modules or health coach)	Physical activity	12 weeks	NR
**Moy [97]**	2016	I: 155<br>C: 84	I: 67 (8.6)<br>C: 66.4 (9.2)	5.2	Veteran patients with COPD	Web-based platform (eg. website with modules or health coach); Wearable device (IMU) (eg. smartwatch)	Physical activity	16 weeks	1. Health related quality of life (St. George's Respiratory Questionnaire Total Score, SGRQ-TS)
**Muller [98]**	2016	I: 22<br>C: 21	I: 63.6 (4.6)<br>C: 62.9 (4.5)	74	Older adults	Text messages	Physical activity	12 weeks	1. Exercise frequency at 12 weeks<br>2. Exercise frequency at 24 weeks
**Munro [99]**	2023	I: 13<br>C: 6	NR	42	People with bowel stoma who have parastomal hernia/bulge	Web-based platform (eg. website with modules or health coach); Video calls (eg. zoom); Other: Email	Physical activity	12 weeks	Feasibility<br>1. Fidelity<br>2. Adherence<br>3. Acceptability<br>4. Safety
**Murphy [100]**	2018	I: 38<br>C: 19	I: 64.8 (8)<br>C: 60.7 (8.5)	76.1	Patients with knee osteoarthritis	Web-based platform (eg. website with modules or health coach)	Physical activity; Mental wellness	6 weeks	1. Physical function (Western Ontario and McMaster Universities Osteoarthritis Index - Physical Function, WOMAC-PF)
**Nahm [47]**	2010	I: 125<br>C: 120	NR	78.4	Older adults	Web-based platform (eg. website with modules or health coach)	Physical activity; Nutrition ; Other: Osteoporosis, falls/hip fractures	2 weeks	NR
**Nelligan [101]**	2021	I: 103<br>C: 103	I: 60.3 (8.2)<br>C: 59 (8.5)	61.2	Patients with knee osteoarthritis	Web-based platform (eg. website with modules or health coach); Text messages	Physical activity	24 weeks	1. Overall average knee pain (Numeric Rating Scale)<br>2. Physical function (WOMAC)
**Okpara [102]**	2023	I: 35<br>C: 35	NR	77.6	Older adults with frailty	Video calls (eg. zoom)	Physical activity; Nutrition ; Mental wellness	12 weeks	NR

**Park [103]**	2020	I: 23<br>C: 21	I: 70.5 (9.4)<br>C: 65.1 (11.1)	21.4	Patients with COPD	Phone calls ; Video calls (eg. zoom)	Physical activity; Nutrition ; Mental wellness ; Other: Smoking cessation, comorbidity management	24 weeks	1. Self-care behaviour
**Peacock [104]**	2020	I: 134<br>C: 70	I: 64 (6)<br>C: 63 (6)	36	Patients at medium/high risk of Cardiovascular Disease or Type II Diabetes Mellitus	Mobile app ; Text messages	Physical activity	12 months	Device-based assessment of physical activity at 12 months:<br>1. Sedentary time (mins/day)<br>2. MVPA (mins/day)<br>3. MVPA10 (MVPA in bouts of at least 10 min, mins/day)<br>4. Vigorous10 (vigorous physical activity in bouts of at least 10 min, mins/day)
**Poppe[2] [105]**	2019	I: 36<br>C: 18	I: 61.6 (8.1)<br>C: 64.9 (8.6)	37	Adults with Type II Diabetes Mellitus	Mobile app ; Wearable device (IMU) (eg. smartwatch)	Physical activity	5 weeks	1. Total physical activity (mins/day)<br>2. MVPA (mins/day)<br>3. Total sitting time (mins/day)
**Porter [106]**	2018	I: 18<br>C: 22	NR	70	Cancer survivors and partners	Video calls (eg. zoom)	Physical activity	24 weeks	Acceptability<br>1. Helpful in increasing PA (patient)<br>2. Helpful in improving communication (patient)<br>3. Recommend to others (patient)
**Prieto-Moreno [107]**	2024	I: 55<br>C: 55	I: 79.6 (7.1)<br>C: 80.1 (7.7)	68.2	Older adults with a hip fracture	Web-based platform (eg. website with modules or health coach); Mobile app	Physical activity; Nutrition ; Other: Medications	12 weeks	1. SPPB at 3 months<br>2. SPPB at 12 months
**Redfern [108]**	2020	I: 486<br>C: 448	I: 66.8 (8.4)<br>C: 68.4 (7.8)	23.3	Patients with or at risk of Cardiovascular Disease	Mobile app	Physical activity; Nutrition	52 weeks	1. Adherence to guideline-recommended medications (≥80% of days covered for blood pressure (BP) and statin medications)
**Rees-Punia [109]**	2022	I: 45<br>C: 40	I: 62.4 (7.3)<br>C: 59.3 (7.4)	94.1	Survivors of physical inactivity-related cancers (Stage I or II breast, colon, endometrium, kidney, or bladder cancer)	Web-based platform (eg. website with modules or health coach); Other: emails	Physical activity	12 weeks	1. Feasibility (Number of participants logged into website at least once)<br>2. Acceptability (Mean rating of motivation, scale 1-5)<br>3. Acceptability (Enjoyment of website, scale 1-5)<br>4. Usability (Mean scores on system usability scale)

**Richard [110]**	2019	I: 1389<br>C: 1335	I: 69 (4.4)<br>C: 69 (4.4)	47.6	Community dwelling older patients at risk of Cardiovascular Disease	Web-based platform (eg. website with modules or health coach)	Physical activity; Nutrition ; Other: Smoking cessation, BP, cholesterol, weight, diabetes	8 weeks	1. Change from baseline to 18 months in systolic BP<br>2. Change from baseline to 18 months in low-density lipoprotein (LDL) cholesterol<br>3. Change from baseline to 18 months in BMI
**Roh [111]**	2022	I: 24<br>C: 25	I: 69.7 (NR)<br>C: 76.9 (NR)	65	Older adults without dementia	Mobile app	Physical activity; Nutrition ; Mental wellness	8 weeks	Brain health behaviour:<br>1. Physical activity change- moderate metabolic equivalent of task (MET-measured by the Global Physical Activity Questionnaire, GPAQ)<br>2. Physical activity- Vigorous MET (GPAQ)<br>3. Cognitive activity change (Cognitive activity score)<br>4. Diet activity change (Mediterranean-DASH (Dietary approaches to stop hypertension) Intervention for Neurodegenerative Delay Diet score)
**Rowley [112]**	2019	I: 119<br>C: 51	I: 67.9 (6.8)<br>C: 66.1 (4.9)	79.5	Inactive older adults	Web-based platform (eg. website with modules or health coach); Wearable device (IMU) (eg. smartwatch)	Physical activity	12 weeks	1. Step count (steps/day)
**Silva [113]**	2023	I: 38<br>C: 35	I: 63.3 (6.7)<br>C: 64.9 (7.6)	12.3	Patients with both Peripheral Artery Disease and Intermittent Claudication	Mobile app ; Phone calls	Physical activity	24 weeks	1. Pain-free walking distance (PFWD) at 6 months<br>2. Functional walking distance (FWD) at 6 months<br>3. Maximal walking distance (MWD) at 6 months<br>4. 6MWD at 6 months
**Stahl [114]**	2020	I: 36<br>C: 9	NR	NR	Bereaved older adults at high risk of depression	Mobile app ; Phone calls	Physical activity; Nutrition ; Mental wellness	12 weeks	NR
**Sun [115]**	2024	I: 34<br>C: 34	NR	55.6	Older adults with hypertension	Web-based platform (eg. website with modules or health coach)	Physical activity; Nutrition ; Other: Medications, Blood pressure	12 weeks	1. Systolic BP<br>2. Diastolic BP<br>3. Exercise adherence (ratio of actual weekly exercise time meeting the prescribed intensity to the total prescribed weekly exercise time)- MET-min/week

									4. Dietary adherence (assessed using the weekly average score of the simplified DASH grading diet index score)- MET-min/week
**Talboom-Kamp [116]**	2017	I: 178<br>C: 37	I: 66.8 (10.6)<br>C: 64.1 (5.7)	47.9	COPD patients	Web-based platform (eg. website with modules or health coach); Text messages ; Phone calls ; Other: Emails	Physical activity	60 weeks	1. Sessions completed<br>2. Session time (mins)<br>3. Total services per user<br>4. Total services per session per user
**Taraldsen [117]**	2020	I: 127<br>C: 63	I: 66.3 (2.3)<br>C: 66.4 (2.7)	52.2	Older adults	Mobile app ; Wearable device (IMU) (eg. smartwatch)	Physical activity	24 weeks	1. Late-Life Function and Disability Index (LLFDI) disability frequency<br>2. Late-Life Function and Disability Index (LLFDI) function<br>3. Behavioural complexity
**Thomsen [118]**	2017	I: 75<br>C: 75	I: 59.7 (10.7)<br>C: 59.5 (12.7)	81	Patients with rheumatoid arthritis	Text messages	Physical activity	16 weeks	1. Change in daily sitting time (ActivPAL)
**Tomita [119]**	2008	I: 16<br>C: 24	I: 74.2 (9.7)<br>C: 77.5 (7.4)	67.5	Patients with Heart Failure	Web-based platform (eg. website with modules or health coach)	Physical activity; Nutrition ; Mental wellness ; Other: Smoking	52 weeks	1. Adherence to program (mean frequency of visits to intervention website per month for all intervention participants)<br>2. Adherence to program (recorded daily vital signs and health activities for all int participants- mean).
**Uemura [120]**	2024	I: 15<br>C: 14	I: 73.9 (3.9)<br>C: 69.4 (3.2)	34.5	Older adults	Wearable device (IMU) (eg. smartwatch); Video calls (eg. zoom)	Physical activity; Nutrition	12 weeks	Feasibility<br>1. Participant retention<br>2. Adherence to the intervention
**VanBakel [121]**	2023	I: 108<br>C: 104	I: 63 (10)<br>C: 64 (10)	23	Patients with Coronary Heart Disease completing cardiac rehabilitation	Mobile app ; Wearable device (IMU) (eg. smartwatch); Phone calls	Physical activity	12 weeks	1. Change in device-based sedentary time between pre- to post-rehabilitation (hours/day)
**VandenHelder [122]**	2020	I: 146<br>C: 101	I: 71.5 (6.4)<br>C: 72.8 (6.5)	71	Older adults	Mobile app ; Video calls (eg. zoom)	Physical activity; Nutrition	24 weeks	1. Physical performance (modified Physical Performance Test, m-PPT)
**VanderVelde [123]**	2021	I: 45<br>C: 41	I: 59 (15)<br>C: 63 (12.6)	49	Patients scheduled for a major elective surgery	Mobile app	Physical activity; Nutrition ; Other: Smoking	NR[3]	Usability (system usability scale)<br>1. Overall usability<br>2. Learnability

									3. Efficiency<br>4. Satisfaction
**VanDyck [124]**	2016	I: 120<br>C: 169	I: 63.1 (2.2)<br>C: 63.2 (2)	52.8	Older Adults	Web-based platform (eg. website with modules or health coach)	Physical activity	5 weeks	NR
**VanDyck [125]**	2019	I: 34<br>C: 38	I: 70.8 (4.1)<br>C: 70.9 (4.1)	49	Older Adults	Web-based platform (eg. website with modules or health coach)	Physical activity	5 weeks	NR
**Vluggen [126]**	2021	I: 234<br>C: 244	I: 59.4 (7.1)<br>C: 60.9 (6.3)	32.4	Patients with Type II Diabetes	Web-based platform (eg. website with modules or health coach)	Physical activity; Nutrition	24 weeks	1. Composition score of changes (composed of changes in physical activity levels, caloric intake from unhealthy snacks, oral hypoglycemic agents, and insulin therapy adherence)
**Webb [127]**	2023	I: 195<br>C: 194	I: 60.6 (NR)<br>C: 61.3 (NR)	96.5	Patients with musculoskeletal condition	Web-based platform (eg. website with modules or health coach); Other: Email	Physical activity	12 weeks	1. Self-reported physical activity (Active Lives Short Measure)
**Yamada [128]**	2023	I: 52<br>C: 47	I: 83.8 (6.7)<br>C: 81.6 (6.1)	72.7	Continuing care retirement community residents	Web-based platform (eg. website with modules or health coach)	Physical activity	16 weeks	1. Participants' mean daily activity using tablet computers every 4 weeks for the following 16 weeks. Daily activity was counted as the number of responses recorded on tablet computers.
**Yardley [129]**	2007	I: 144<br>C: 136	I: 77.5 (6.8)<br>C: 77.0 (7.4)	66	Older adults	Web-based platform (eg. website with modules or health coach)	Physical activity	NR	NR
**Zamanillo-Campos [130]**	2023	I: 96<br>C: 111	I: 63 (10)<br>C: 61 (12)	34.8	Patients with Type II Diabetes	Text messages	Physical activity; Nutrition ; Mental wellness ; Other: Medications, Sexual health, Smoking, Caregiver support, Foot care	12 weeks	NR
**Zamorano [131]**	2021	I: 40<br>C: 40	I: 61.5 (NR)<br>C: 61.5 (NR)	100	Endometrial cancer survivors with obesity	Text messages	Physical activity; Nutrition	24 weeks	1. Weight loss at 6 months

**Zhang [48]**	2024	I: 1038<br>C: 1034	I: 61.4 (7.1)<br>C: 61.6 (6.9)	44.9	Patients with Type II Diabetes and a glycated haemoglobin (HbA1c) ≥7%	Mobile app	Physical activity; Nutrition ; Other: Monitoring blood glucose and BP, Medications, Accessing healthcare, Smoking	104 weeks	1. Difference in proportion of patients achieving at least two "ABC" goals defined as any two of the following: HbA1c < 7.0%, both systolic/diastolic BP < 140/80 mmHg and LDL-cholesterol < 100 mg/dl or 2.6 mmol/L) at 24 months
**Zhao [132]**	2022	I: 24<br>C: 24	I: 65.8 (3.2)<br>C: 65.6 (4.2)	63.2	Older adults	Other: Nintendo switch	Physical activity	12 weeks	NR
**Zheng [133]**	2019	I: 411<br>C: 411	I: 56.3 (9.3)<br>C: 56.6 (9.7)	14.1	Patients with Coronary Heart Disease but no Diabetes	Text messages	Physical activity; Other: Medications, Smoking	24 weeks	1. Change in systolic BP from baseline to 6 months.

[1]Only considered timepoint at 3mo as 6mo timepoint did not have a true control.

[2] This study included two RCTs. Only RCT 1 is used here.

[3]Unclear as the intervention length is tailored according to each participants' surgery date.
