# Supplementary material for: Behaviour change techniques in eHealth interventions for older, frail, or sarcopenic adults: A systematic review and meta-analysis
Source: Digit Health. 2026 Jul 28;12:20552076261473804. doi: 10.1177/20552076261473804 (PMC13420075; doi:10.1177/20552076261473804)
Supplement: Supplemental material - Behaviour change techniques in eHealth interventions for older, frail, or sarcopenic adults: A systematic review and meta-analysis [file sj-pdf-4-dhj-10.1177_20552076261473804.pdf]

S4 Fig. Risk of Bias judgements for each included study.

	D1	D2	D3	D4	D5	Overall
Arbillaga-Etxarri 2018	+	+	+	+	+	+
Azar 2016	+	+	+	+	+	+
Baez 2017	+	+	+	+	!	!
Bailey 2024	+	+	+	!	-	-
Beckie 2024	+	!	+	+	+	+
Bennell 2020	+	+	+	+	+	+
Bentley 2020	+	-	-	-	-	-
Benzo 2022	!	+	+	+	+	+
Bickmore 2013	+	!	!	+	+	!
Bisson 2021	+	!	+	+	!	!
Blair 2021	+	!	+	+	+	+
Bonn 2024	+	+	+	+	+	+
Bowen 2022	+	!	+	+	!	!
Bronas 2024	+	+	+	+	+	+
Cadmus-Bertram 2015	+	+	+	+	!	!
Cadmus-Bertram 2016	+	+	+	+	!	!
Chan 2020	+	+	+	+	+	+
Dale 2015	+	+		!	+	!
Engelen 2020		-	+	+	!	-
Evans 2021	+	+	+	+	!	!
Fanning 2020	+	+	+	+	+	+
Fanning 2023	+	!	+	-	-	-
Fanning 2022	+	+	+	+	+	+
Finlay 2020	+	+	+	+	+	+
Frederix 2017	+	+	+	+	+	+
Freer 2024	+	!	+	+	+	!
Gerber 2024	+	+	+	+	+	+
Guillaumier 2022	+	+	+	+	+	+
Hawley-Hague 2023	+	+	+	+	+	+

+	Low risk
!	Some concerns
-	High risk

**D1**	Randomisation process
**D2**	Deviations from the intended interventions
**D3**	Missing outcome data
**D4**	Measurement of the outcome
**D5**	Selection of the reported result

**Hou 2024**	!	!	+	+	!	!
**Ilie 2023**	+	+	+	+	+	+
**Jennings 2023**	+	!	+	!	+	!
**Jiang 2020**	+	+	+	+	+	+
**Kenfield 2019**	+	+	+	+	+	+
**Kenfield 2021**	+	+	+	+	+	+
**Kim 2013**	-	!	+	!	+	-
**King 2020**	+	+	+	+	+	+
**Kroesen 2024**	+	+	+	+	+	+
**Kwan 2020**	+	+	+	+	!	!
**Lally 2024**	+	+	+	+	+	+
**Langlais 2021**	+	+	+	+	-	-
**Laslovich 2020**	+	+	+	+	!	!
**Lindsay 2009**	!	+	+	+	-	-
**Little 2024**	+	!	+	+	+	!
**Lyons 2017**	+	+	+	+	+	+
**Maddison 2019**	+	+	+	+	+	+
**McCourt 2023**	-	!	+	+	+	-
**Mouton 2015**	!	!	+	-	!	-
**Moy 2016**	+	-	+	+	+	-
**Muller 2016**	+	+	+	!	+	!
**Munro 2023**	+	!	!	+	+	!
**Murphy 2018**	+	+	+	+	!	!
**Nahm 2010**	+	+	+	+	-	-
**Nelligan 2021**	+	+	+	+	+	+
**Okpara 2023**	+	+	+	+	+	+
**Park 2020**	+	!	+	+	!	!
**Peacock 2020**	+	+	+	+	+	+
**Poppe 2019**	+	-	+	!	+	-
**Porter 2018**	+	+	+	!	-	-

Prieto-Moreno 2024	+	+	+	+	+	+
Redfern 2020	+	+	+	+	+	+
Rees-Punia 2022	+	-	+	+	!	-
Richard 2019	+	+	+	+	+	+
Roh 2022	!	+	+	+	+	!
Rowley 2019	!	+	+	+	!	!
Silva 2023	+	+	+	+	+	+
Stahl 2020	+	+	+	+	+	+
Sun 2024	+	+	+	-	+	-
Talboom-Kamp 2017	+	+	-	+	+	-
Taraldsen 2020	+	+	+	-	+	-
Thomsen 2017	+	+	+	+	+	+
Tomita 2009	!	+	+	+	!	!
Uemura 2024	+	+	+	+	+	+
vanBakel 2023	+	!	+	+	+	!
vandenHelder 2020	+	+	+	+	+	+
vanderVelde 2021	+	+	+	+	+	+
VanDyck 2019	-	+	+	+	!	-
VanDyck 2016	+	-	-	-	+	-
Vluggen 2021	+	+	+	+	+	+
Webb 2023	!	!	+	!	+	!
Yamada 2023	+	+	+	+	!	!
Yardley 2007	+	+	+	+	-	-
Zamanillo-Campos 2023	+	+	+	+	+	+
Zamorano 2021	+	-	-	-	+	-
Zhang 2024	+	!	+	+	+	!
Zhao 2022	+	!	+	+	!	!
Zheng 2019	+	+	+	+	+	+
