# Supplementary figures and images for: Behaviour change techniques in eHealth interventions for older, frail, or sarcopenic adults: A systematic review and meta-analysis
Source: Digit Health. 2026 Jul 28;12:20552076261473804. doi: 10.1177/20552076261473804 (PMC13420075; doi:10.1177/20552076261473804)

**S5 Fig.** Funnel plot of study effect sizes and precision.

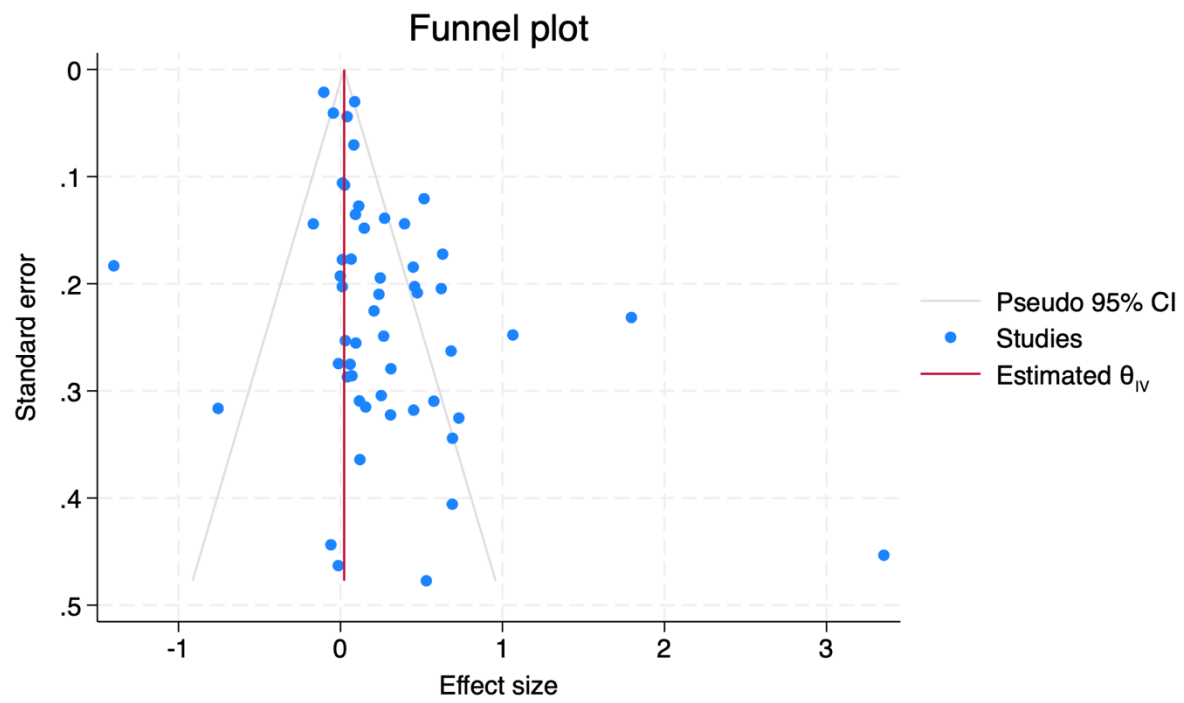

Supplement: Supplemental material - Behaviour change techniques in eHealth interventions for older, frail, or sarcopenic adults: A systematic review and meta-analysis [file sj-pdf-5-dhj-10.1177_20552076261473804.pdf]
