# Supplementary material for: Behaviour change techniques in eHealth interventions for older, frail, or sarcopenic adults: A systematic review and meta-analysis
Source: Digit Health. 2026 Jul 28;12:20552076261473804. doi: 10.1177/20552076261473804 (PMC13420075; doi:10.1177/20552076261473804)
Supplement: Supplemental material - Behaviour change techniques in eHealth interventions for older, frail, or sarcopenic adults: A systematic review and meta-analysis [file sj-pdf-6-dhj-10.1177_20552076261473804.pdf]

S6 Table. Egger's test for small-study effects.

Std_Eff	Coefficient	Std. err	t	p >|t|	95% conf. interval
**slope**	-0.08	0.39	-1.91	0.06	-0.15 to 0.00
**bias**	1.57	0.41	3.81	0.00	0.74 to 2.39
