# Supplementary material for: Behaviour change techniques in eHealth interventions for older, frail, or sarcopenic adults: A systematic review and meta-analysis
Source: Digit Health. 2026 Jul 28;12:20552076261473804. doi: 10.1177/20552076261473804 (PMC13420075; doi:10.1177/20552076261473804)
Supplement: Supplemental material - Behaviour change techniques in eHealth interventions for older, frail, or sarcopenic adults: A systematic review and meta-analysis [file sj-pdf-7-dhj-10.1177_20552076261473804.pdf]

S7 Table. Subgroup analyses by RoB-2 classification (n= 53 studies).

**Risk of bias studies**	**SMD (95% CI)**
Low risk	0.13 (0.01, 0.25)
Some risk	0.37 (0.21, 0.53)
High risk	0.39 (-0.09, 0.88)
Between-groups p-value	p=0.05
